# Lignin biosynthesis pathway repressors in gymnosperms: differential repressor domains as compared to angiosperms

**DOI:** 10.48130/forres-0024-0029

**Published:** 2024-09-19

**Authors:** Sonali Sachin Ranade, María Rosario García-Gil

**Affiliations:** Umeå Plant Science Centre (UPSC), Department of Forest Genetics and Plant Physiology, Swedish University of Agricultural Sciences, 901 83 Umeå, Sweden

**Keywords:** Conifers, Gymnosperms, Lignin-repressors, MYB, Norway spruce, Scots pine

## Abstract

Lignin is a polyphenolic polymer present in the cell walls of specialized plant cell types in vascular plants that provides structural support and plays a major role in plant protection. The lignin biosynthesis pathway is regulated by transcription factors from the MYB (myeloblastosis) family. While several MYB members positively regulate lignin synthesis, only a few negatively regulate lignin synthesis. These lignin suppressors are well characterized in model plant species; however, their role has not been fully explored in gymnosperms. Lignin forms one of the major hurdles for the forest-based industry e.g. paper, pulp, and biofuel production. Therefore, the detailed mechanisms involved in the regulation of lignin synthesis are valuable, especially in conifers that form the major source of softwood for timber and paper production. In this review, the potential and differential domains present in the MYB suppressors in gymnosperms are discussed, along with their phylogenetic analysis. Sequence analysis revealed that the N-terminal regions of the MYB suppressor members were found to be conserved among the gymnosperms and angiosperms containing the R2, R3, and bHLH domains, while the C-terminal regions were found to be highly variable. The typical repressor motifs like the LxLxL-type EAR motif and the TLLLFR motif were absent from the C-terminal regions of MYB suppressors from most gymnosperms. However, although the gymnosperms lacked the characteristic repressor domains, a R2R3-type MYB member from *Ginkgo* was reported to repress the lignin biosynthetic pathway. It is proposed that gymnosperms possess unique kinds of repressors that need further functional validation.

## Introduction

Lignin is one of the most abundant secondary metabolites present in the cell walls of specialized plant cell types in vascular plants^[1]^. Lignin is an organic polyphenolic polymer that is formed by the polymerization of three monolignols – p-coumaryl alcohol, coniferyl alcohol, and sinapyl alcohol, that produce p-hydroxyphenyl (H), guaiacyl (G), and syringyl (S) subunits, respectively. Gymnosperms lack the S-lignin subunits and have relatively higher lignin content compared to the angiosperm woody species^[2]^. Lignin provides structural support, transports water and minerals, and protects the plant from pathogens, thereby acting as a barrier^[3]^. However, lignin forms one of the major hurdles for the forest-based industry e.g. paper, pulp, and biofuel production^[4]^. For example, lignin negatively affects paper quality where the presence of lignin causes discoloration of the paper and weakens it. The degradation of lignin is difficult as it is a complex polymer, therefore modification or pretreatment of lignin is required to make the wood suitable for biofuel production^[5]^. Hence, the detailed mechanisms involved in the regulation of lignin synthesis are valuable for the industry. Conifers form the major source of softwood for timber and paper production, and they are especially preferred for pulp because of the long fibers in their wood^[6]^. Even so, while the lignin biosynthetic pathway is well studied in model plants like *Arabidopsis thaliana* (*Arabidopsis*) and other angiosperm woody trees, this research area remains relatively unexplored in gymnosperms.

The lignin biosynthesis pathway is regulated by a complex network of transcription factors from the MYB (myeloblastosis) family that either positively or negatively controls lignin synthesis^[7]^. In this review, MYB members were referred to that activate or suppress lignin synthesis as MYB activators or MYB suppressors/repressors, respectively. This review focuses on the potential and differential domains present in the MYB suppressors in gymnosperms along with their phylogenetic analysis. The details of all the sequences included for the domain and phylogenetic analysis are included in Supplemental Table S1.

## MYB transcription factors involved in the lignin biosynthesis pathway

MYB family members are functionally diverse, and apart from regulating the lignin biosynthesis pathway they also control various processes involved in plant growth and development^[8,9]^. The N-terminal region of the MYB proteins is highly conserved containing the MYB repeats (R) involved in DNA binding; there are three types of R repeats – R1, R2, and R3. The MYB family in plants is classified into four classes according to the presence of the number of R domain repeats: 1R-MYB or MYB-related (having R1/R2/R3), R2R3-MYB (having R2 and R3), 3R-MYB (having R1, R2 and R3), and 4R-MYB (having R1, two R2 and R1/R2)^[8]^. The C-terminal region of the MYB proteins is highly variable and contains the regulatory domain (activation/suppression domain)^[8]^.

While there are several MYB members that positively regulate lignin synthesis, only a few from the R2R3-MYB class and their homologues in plant species including woody trees, negatively regulate lignin synthesis^[10]^. *Arabidopsis* is the most well studied plant model, where the R2R3-MYB members are well characterized. The R2R3-MYB class in *Arabidopsis* is the largest among the MYB family with 126 members which contains the basic helix-loop-helix (bHLH) domain within the R3 region. The R2R3 class is further classified into 25 subgroups depending on the motifs in the C-terminal region^[9]^. Subgroup 4 of the R2R3-MYB class comprises four members in *Arabidopsis* - MYB3, MYB4, MYB7, and MYB32^[11,12]^. These members contain conserved MYB motifs - LLsrGIDPxT/SHRxI/L (C1 motif) at the end of the R3 repeat and the C2 motif (pdLHLD/LLxIG/S) in the C-terminal regions. The C2 motif harbors the LxLxL-type or DLNxxP-type repression motif (Ethylene-responsive element binding factor-associated Amphiphilic Repression abbreviated as EAR)^[13−15]^. MYB4, MYB7, and MYB32 additionally possess a putative zinc-finger motif (ZF motif, CX_1–2_CX_7–12_CX_2_C) and a conserved GY/FDFLGL motif (part of the C4 motif) in their C-termini region^[14]^. MYB3, MYB4, MYB7, and MYB32 have been demonstrated to function as the transcriptional repressors of phenylpropanoid pathway - lignin biosynthesis pathway and/or the biosynthesis of pigments in *Arabidopsis*^[11,12]^. MYB3 negatively regulates *cinnamate 4-hydroxylase* (*C4H*) that catalyses the second step of the phenylpropanoid pathway leading to lignin and pigment synthesis, while MYB4 can inhibit almost all the enzymes in the lignin synthesis pathway causing a decrease in lignin synthesis^[10,16]^. R2R3-MYB members from other angiosperm species that negatively regulate lignin biosynthesis included in this review are MYB156/MYB221 from populus (*Populus trichocarpa*), EgMYB1 from eucalyptus (*Eucalyptus gunnii*), ZmMYB31/ZmMYB42 from maize (*Zea mays*) and PvMYB4 from switchgrass (*Panicum virgatum*)^[10,17−20]^.

Similar to the repressors, the R2R3-MYB members that act as activators of the lignin biosynthesis pathway possess the conserved R2, R3, and bHLH domains in their N-terminal region, and their C-terminal region is highly variable. But unlike the repressors, where the characteristic repressor motif e.g. EAR has been described, the presence of a specific activator motif has not been described in the R2R3-MYB activators of the lignin biosynthesis pathway in multiple angiosperm species such as *Arabidopsis* (MYB58, MYB63)^[21]^, eucalyptus (EgMYB2)^[22]^ and populus (*Populus tomentosa*, PtoMYB92, PtoMYB216)^[23,24]^. However, a few activator motifs such as SG7 and SG7-2^[9,25]^ have been reported in the C-terminal of R2R3-MYB members belonging to subgroup 7, which positively regulates flavonoid synthesis e.g. in grapevine (VvMYBF1)^[25]^ and *Arabidopsis* (AtMYB12 and AtMYB111)^[9]^.

Both, the repressor and activator R2R3-MYB members, repress or activate the genes from the lignin biosynthesis pathway respectively, by binding to the AC elements (adenosine and cytosine-enriched sequences) present in the promoters of lignin biosynthetic genes^[26]^. Another way in which these R2R3-MYB members function is by interacting with Glabrous 3 (GL3), which is the key element of the MYB-bHLH-WD40 (MBW) complex that regulates the lignin biosynthesis pathway, flavonoid biosynthesis and trichome development in *Arabidopsis*^[27]^. The repressors compete with the activators to bind to GL3 or to the AC elements of promoters, to bring about the repression of the lignin biosynthesis pathway genes^[12,28]^.

Apart from repressors and activators, the R2R3-MYB family comprises members that act as master regulators of cell wall formation, e.g. MYB46 which is a multifaceted R2R3-MYB transcription factor in *Arabidopsis*. MYB46 along with its paralogue MYB83, functions as a master switch for the secondary cell wall biosynthesis that not only mediates the transcriptional network involved in the secondary cell wall formation, but also regulates the genes from the cellulose, hemicellulose and lignin biosynthesis pathways including upstream regulators and downstream targets^[29]^.

## Phylogenetic analysis of MYB repressors of the lignin biosynthesis pathway in gymnosperms

To date, only two studies in gymnosperms have validated the repressor activity of the R2R3-MYB transcription factor in the lignin biosynthetic pathway - GbMYBR1 in *Ginkgo biloba* (*Ginkgo*) and CfMYB5 in Chinese cedar (*Cryptomeria fortunei* Hooibrenk)^[30,31]^. Recently, differential regulation of the *MYBs* (copies of *MYB3* and *MYB4*) that potentially act as suppressors and the variation in lignin synthesis in response to light quality in the Norway spruce (*Picea abies*)^[32]^ and Scots pine (*Pinus sylvestris*)^[33]^ seedlings were reported based on the transcriptomic and Fourier transform infrared (FTIR) analysis. The Scots pine reads in the previous study^[33]^ were aligned to the loblolly pine (*Pinus taeda*) genome (v1.01)^[34]^, therefore the corresponding MYB sequences retrieved from loblolly pine were considered for this review. MYB3 copies from *Picea* were named Pa_AtMYB3-like1, Pa_AtMYB3-like2, and so on, while copies from *Pinus* were named Pt_AtMYB3-like1, Pt_AtMYB3-like2, and so on. A similar naming convention was followed for the MYB4 copies from both conifers. A total of 23 MYB repressors from gymnosperms including eight sequences from Norway spruce, 13 sequences from loblolly pine and one sequence from *Ginkgo* and one sequence from Chinese cedar were recruited for the analysis.

**Figure 1:**
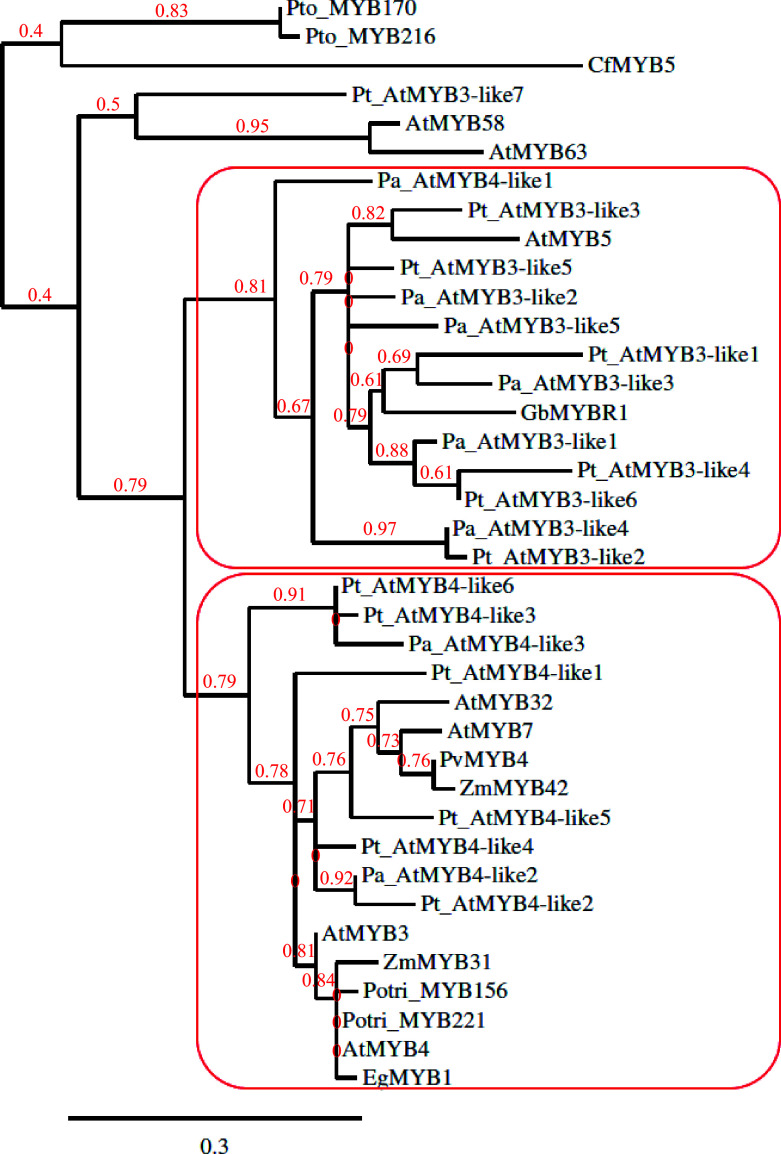
Phylogenetic tree constructed with copies of MYB3-like and MYB4-like from *Picea abies* (Pa) and *Pinus taeda* (Pt) along with GbMYBR1 from *Ginkgo biloba* (Gb); CfMYB5 from *Cryptomeria fortune* (Cf); MYB3, MYB4, MYB5, MYB7, MYB32, MYB58 and MYB63 from *Arabidopsis thaliana* (At); MYB156 and MYB221 from *Populus* *trichocarpa* (Potri); MYB170 and MYB216 from *Populus tomentosa* (Pto); EgMYB1 from *Eucalyptus gunnii* (Eg); ZmMYB31 and ZmMYB42 from *Zea mays* (Zm) and, PvMYB4 from *Panicum virgatum* (Pv).

The earlier phylogenetic analysis suggested GbMYBR1 to be a distinct MYB suppressor closely related to MYB5 from *Arabidopsis*^[30]^ and showed that CfMYB5 was grouped with ZmMYB31 (*Zea mays*), EgMYB1 (*Eucalyptus grandis*) and AtMYB4 (*Arabidopsis*), which inhibited lignin synthesis^[31]^. For the current review, MYB members reported by earlier studies^[11,12,17−20,24,30,32,33,35]^ were included in the phylogenetic tree (Fig. 1), which was constructed using Phylogeny.fr with default settings^[36]^. MYB members from *Arabidopsis* that repress the phenylpropanoid pathway such as MYB3, MYB4, MYB7 and MYB32 (AtMYB3, AtMYB4, MYB7, MYB32) were included in the phylogenetic tree as *Arabidopsis* is the most well-studied model system in plants. CfMYB5 and GbMYBR1 were included in the phylogenetic tree as they are the R2R3-MYB repressor genes from gymnosperms that negatively regulate the lignin biosynthesis pathway^[30,31]^. MYB5 from *Arabidopsis* (AtMYB5) was included in the construction of the phylogenetic tree as some of the MYB members from conifers showed the presence of the provisional MYB5 repressor in the C-terminal domain in the Conserved Domain Database^[37]^ (CDD) search and GbMYBR1 is closely related to MYB5 from *Arabidopsis*^[30]^. The MYB3/MYB4 copies from Norway spruce and Scots pine (corresponding *Pinus taeda* sequences) from earlier studies^[32,33]^, which were proposed to repress lignin synthesis, were included in the phylogenetic tree. R2R3-MYB family members from a few other species that repress lignin biosynthesis were included in the phylogenetic tree, e.g. Potri_MYB156 and Potri_MYB221 from populus; EgMYB1 from eucalyptus; PvMYB4 from switchgrass; ZmMYB31 and ZmMYB42 from maize^[10,17−20]^. PtoMYB170 and PtoMYB216 from *Populus tomentosa* and, AtMYB58 and AtMYB63 from *Arabidopsis*, which positively regulates lignin deposition during the formation of wood^[21,35]^, were included in the phylogenetic tree as an outgroup.

The phylogenetic tree (Fig. 1) shows two distinct sub-clades, one sub-clade that contains all the MYB3-like members from the two conifers (except Pt_AtMYB3-like7) and one MYB4-like member from spruce (Pa_AtMYB4-like3), along with AtMYB5 and GbMYBR1. The other sub-clade includes all the MYB4-like members from the two conifers along with AtMYB3 and AtMYB4 from *Arabidopsis*, and the R2R3-MYB family suppressors from populus, eucalyptus, switchgrass, and maize. Overall, the phylogenetic analysis shows a clear separation of the MYB3-like and MYB4-like R2R3-MYB members from the two conifer species into two groups, where the GbMYBR1 from *Ginkgo* groups with the MYB3-like members. This suggests that the MYB3-like members from conifers and *Ginkgo* may have distinct motifs which differ from the motifs present in the angiosperms. However, the MYB4-like conifer members seem to have motifs that are similar to angiosperm species e.g. many of the MYB4-like conifer members contain the EAR motif, while EAR was detected in only one of the MYB3-like members (Pt_AtMYB3-like1). CfMYB5 from Chinese cedar seems to contain unique motifs compared to all the gymnosperm members included in this study.

## Domains of MYB repressors from the lignin biosynthesis pathway in gymnosperms

**Figure 2:**
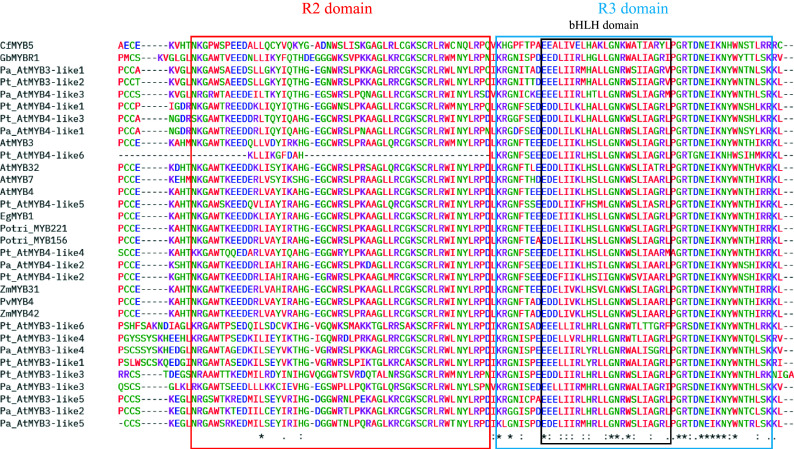
Alignment of N-terminal regions of the lignin repressor MYB members from gymnosperms and angiosperms showing the conserved R2, R3, and bHLH domains: MYB3-like and MYB4-like copies from *Picea abies* (Pa) and *Pinus taeda* (Pt) along with GbMYBR1 from *Ginkgo biloba* (Gb); CfMYB5 from *Cryptomeria fortune* (Cf); MYB3, MYB4, MYB7, and MYB32 from *Arabidopsis thaliana* (At); MYB156 and MYB221 from *Populus* *trichocarpa* (Potri); EgMYB1 from *Eucalyptus gunnii* (Eg); ZmMYB31 and ZmMYB42 from *Zea mays* (Zm) and PvMYB4 from *Panicum virgatum* (Pv).

**Figure 3:**
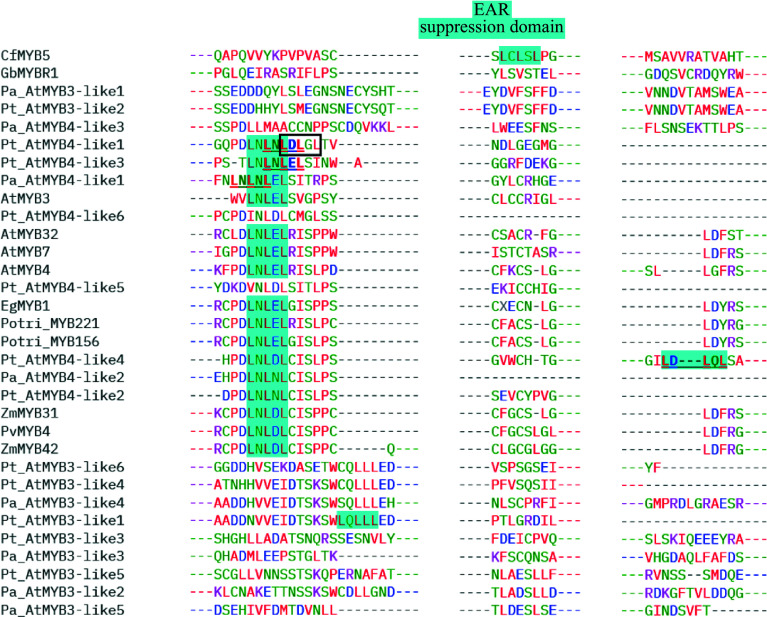
Alignment of partial C-terminal regions of the lignin repressor MYB members from gymnosperms and angiosperms showing the conserved EAR domain (alternative EAR domains in Pt_AtMYB4-like1, Pt_AtMYB4-like3, Pt_AtMYB4-like4, and Pa_AtMYB4-like1 are marked with box and, bold and underlined): MYB3-like and MYB4-like copies from *Picea abies* (Pa) and *Pinus taeda* (Pt) along with GbMYBR1 from *Ginkgo biloba* (Gb); CfMYB5 from *Cryptomeria fortune* (Cf); MYB3, MYB4, MYB7, and MYB32 from *Arabidopsis thaliana* (At); MYB156 and MYB221 from *Populus* *trichocarpa* (Potri); EgMYB1 from *Eucalyptus gunnii* (Eg); ZmMYB31 and ZmMYB42 from *Zea mays* (Zm) and PvMYB4 from *Panicum virgatum* (Pv).

Alignments of MYB repressors from the gymnosperms and angiosperms species are included in the Supplemental information (Supplemental Figs S1–S5). GbMYBR1 shows distinct sequence characteristics; it has low identity with characterized MYB4 repressors from *Arabidopsis* and other angiosperm species. Although the characteristic domains of the R2R3-type repressors e.g. C1, C2, ZF, and C4 motifs and, the typical repressors motifs like the LxLxL-type EAR motif and the TLLLFR motif are absent in GbMYBR1 from the C-terminal, GbMYBR1 has the R2 and R3 domain in the N-terminal region including the conserved bHLH-binding motif (Fig. 2)^[30]^. CfMYB5 from Chinese cedar contains the conserved R2 and R3 domains in the N-terminal like *Ginkgo*, however, the study did not report any typical R2R3-type suppressor domain in its C-terminal^[31]^. The current sequence analysis reports the presence of the EAR suppression domain (LCLSL) in the C-terminal region of CfMYB5 (Fig. 3, Supplemental Fig. S5), which is novel.

Copies or homologs of *MYB3* and *MYB4* were detected to be differentially regulated under shade (Low Red : Far-red) in Norway spruce and Scots pine and these MYB copies were proposed to repress the lignin synthesis as their down-regulation correlated with enhanced lignin synthesis^[32,33]^. Alignments^[38]^ performed with the different copies of MYB repressors reported by earlier studies^[8,30,32,33]^ show that the sequences are well conserved in gymnosperms and angiosperms in the N-terminal regions (R2, R3 along with the bHLH binding motif) but not in the C-terminal region (Fig. 2, Supplemental Figs S1−S5) which are in accordance with the previous findings. The C-terminal regions (with the C1, C2, ZF, and C4 motifs) are the most variable regions within the different conifer MYB members (Supplemental Figs S1−S4) which agrees with the findings in *Arabidopsis*^[8,9]^. The alignment of partial C-terminal regions of MYB repressors from gymnosperms and angiosperms (Fig. 3) show that most MYB3/MYB4 copies from both conifers lack the classical LxLxL-type EAR motif. It is worth noting that generally monocots possess the LNLDL motif and dicots have the LNLEL motif, while the conifers show the presence of both LNLDL and LNLEL, in addition to four more patterns – LNLNL, LDLGL, LDLQL, and LQLLL (Fig. 3). In Pt_AtMYB4-like1, Pt_AtMYB4-like3, Pt_AtMYB4-like4, and Pa_AtMYB4-like1, either LNLNL/LNLDL/LDLGL, LNLNL/LNLEL or LNLDL/LDLQL or LNLNL/LNLEL could function as a potential repressor, respectively (alternative EAR domains are marked with a box and, bold and underlined for Pt_AtMYB4-like1, Pt_AtMYB4-like3, Pt_AtMYB4-like4, and Pa_ AtMYB4-like1 in Fig. 3). It is also possible that LNLNLDLGL and LNLNLEL could function as a unique type of lignin repressor in conifers. CfMYB5 from Chinese cedar also shows the presence of a new pattern of the EAR motif (LCLSL), which was not described previously in this species by earlier investigations and is not represented in the angiosperms. None of the gymnosperm MYB members contain the conserved TLLLFR motif or the GY/FDFLGL motif, which are essential for the repressor activity of the transcription factors (Supplemental Figs S4 & S5)^[12,14]^. This is similar to the unique kind of R2R3-MYB-type repressor reported recently in *Ginkgo* (GbMYBR1)^[30]^. Similar to GbMYBR1, all the MYB3/MYB4 copies in both the conifer species contain the bHLH binding motif in the R3 region that could potentially be involved in the repression mechanism^[30]^.

Except for two MYB members in *Pinus* (Pt_AtMYB3-like7 and Pt_AtMYB4-like3), all the other MYB sequences in both conifers possess either the EAR repressor motif in the C-terminal (Fig. 3) and/or the putative repressor domain PLN03212 in the N-terminal region (Supplemental Table S1). The PLN03212 domain was detected in the motif search using CDD^[37]^ with a very low E-value. The PLN03212 domain is the provisional repressor domain for the transcription factor MYB5 in *Arabidopsis*, but this domain has not been functionally characterized. MYB5 represses the flavonoid pathway, regulates mucilage synthesis, and plays a role in the development of the seed coat and trichome morphogenesis^[39]^. The PLN03212 domain is also present in GbMYBR1, but its potential function in the process of repression has not been reported^[30]^. The PLN03091 was yet another domain that was detected in the searches with CDD with a very low E-value in MYB suppressors from both conifers, similar to lignin repressors of other plant species (Supplemental Table S1). The PLN03091 is denoted as a provisional hypothetical protein in the CDD.

It is proposed that conifers contain different types of MYB3/MYB4-like repressors, some of which contain the classical repressor motifs and others without the repressor motifs (e.g. LxLxL) analogous to that of GbMYBR1*.* Similar to GbMYBR1, the MYB3/MYB4-like repressors detected in Norway spruce and Scots pine^[32,33]^ might have distinct sequence characteristics or motifs, whose potential functional characterisation needs further validation. These repressors and their mode of regulation especially regarding the defense and phenylpropanoid pathways might be unique to conifer species, which also needs further characterization.

## MYB repressors from gymnosperms are functionally different than angiosperms

The repressors from the MYB family are not fully explored in gymnosperms unlike the well-studied model plants e.g. *Arabidopsis*. *GbMYBR1* from *Ginkgo* is mainly expressed in young leaves, although its expression was detected in the roots, stem and fruits; the expression level of *GbMYBR1* in young leaves were more than 12-fold higher than the levels in roots or stems. GbMYBR1 is a unique kind of R2R3-MYB-type repressor that lacks the characteristic repressor motifs e.g. EAR motif and the TLLLFR motif from the C-terminal region, yet it suppresses the lignin biosynthesis pathway. Overexpression of *GbMYBR1* in *Arabidopsis* represses lignin synthesis specifically through down-regulation of the key lignin biosynthesis pathway gene – *hydroxycinnamoyl CoA: shikimate hydroxycinnamoyl transferase* (*HCT*) which encodes the enzyme that catalyzes the rate-limiting step of the lignin biosynthesis pathway. In addition, GbMYBR1 down-regulates a few other genes from the lignin biosynthesis pathway including *phenylalanine ammonia-lyase* (*PAL*), *4-coumarate: CoA ligase* (*4CL*) and *cinnamyl alcohol dehydrogenase* (*CAD*). *GbMYBR1* overexpression also reduces the pathogen resistance by significantly down-regulating a great number of defence-related genes in the transgenic *Arabidopsis*. Moreover, the transgenic *Arabidopsis* overexpressing *GbMYBR1* was more susceptible to bacterial infection as compared to the wild type. However, the regulatory process of the *GbMYBR1* is entirely different from *Arabidopsis*; the lignin synthesis suppression by GbMYBR1 is not only more specific compared to the MYB repressors in *Arabidopsis* but the mode of action of GbMYBR1 to repress lignin synthesis is different from *Arabidopsis*^[30]^. The GbMYBR1 mode of action is mediated through direct and specific interaction with GL3 to compete against the interaction of GL3 with MYB activators, leading to the suppression of lignin synthesis^[30]^. For example, in *Arabidopsis*, the MYB4 that represses the lignin biosynthesis pathway, physically interacts not only with GL3 but also with other bHLH cofactors (e.g. TT8 and EGL3) to bring about the suppression^[40]^. Thus, the interaction of *Arabidopsis* repressor MYB with bHLH cofactors is not as specific as for GbMYBR1. In addition, overexpression of *GbMYBR1* led to the down-regulation of *HCT* – a key gene from the lignin biosynthesis pathway along with only a few other genes from the lignin biosynthesis pathway in contrast to other angiosperm species where expression of multiple genes was affected along with reduced lignification as a result of the overexpression of *MYB* that acts as a repressor^[30]^. Thus, the suppression of GbMYBR1 on lignin biosynthesis is more specific than for the other repressor MYBs in *Arabidopsis*. Su et al. proposed the working model of GbMYBR1 and presented the details on the regulatory mechanism of *GbMYBR1* in transgenic *Arabidopsis*^[30]^, yet whether *GbMYBR1* directly regulates the genes from lignin biosynthetic pathway in *Ginkgo* needs to be further explored and validated*.*

CfMYB5 from Chinese cedar which negatively regulates the lignin biosynthesis is a nucleus-localized protein that is expressed at higher levels in the stem as compared to the needle, bud, male cone, and root^[31]^. The repressor activity of *CfMYB5* was demonstrated from the analysis of its expression patterns; overexpression of *CfMYB5* in the transgenic lines correlated with the decrease in expression of the key genes involved in the lignin biosynthesis pathway (*HCT*, *PAL*, *4CL*, and *CAD*) along with a decrease in secondary cell wall formation which involves the deposition of both lignin and cellulose. Thus, *CfMYB5* suppression was not specific only for lignin synthesis.

## Regulation of lignin MYB suppressors by light quality in conifers – MYB expression and lignin synthesis

*MYB4* has been demonstrated to respond to light quality in *Arabidopsis*; for example, UV-B irradiation down-regulates *MYB4*^[13]^. As the MYB repressor is involved in the negative regulation of the lignin biosynthesis pathway, its down-regulation leads to higher lignin synthesis. In Norway spruce, down-regulation of two copies of *MYB3* under shade (Low Red : Far-red ratio) in the northern populations correlated with higher lignin synthesis in the case of north vs south comparisons^[32]^ (Supplemental Table S1). While none of the repressors were detected to be differentially regulated under shade in the southern population, an equal number of repressors (*MYB3/MYB4*) were found to be up-regulated and down-regulated (*p*-value > 0.05) in the northern population of Norway spruce^[32]^. Nevertheless, *MYB3/MYB4* gene expression in Scots pine was not fully in favour of higher lignin synthesis under shade^[33]^. The Scots pine reads in a previous study^[33]^ were aligned to the loblolly pine (*Pinus taeda*) genome (v1.01)^[34]^, therefore the corresponding MYB sequences retrieved from loblolly pine were considered for this review, as mentioned previously. Therefore, it is important to note that the *Pinus taeda* information in Supplemental Table S1 corresponds to Scots pine. An equal numbers of repressors (*MYB3/MYB4*) were found to be up-regulated and down-regulated (*p*-value > 0.05) respectively in the southern and northern Scots pine population under shade (Supplemental Table S1). In the case of north vs south comparisons, four *MYB* members were found to be up-regulated in the northern Scots pine population as compared to the southern population under shade, suggesting higher lignin synthesis in the southern population^[33]^. The analysis for genes that positively regulate the lignin biosynthesis in these studies suggest an equal number of genes being up-regulated and down-regulated under shade in both the conifer species^[32,33]^. However, the FTIR spectroscopic data confirmed higher synthesis of lignin in response to shade as compared to the sun conditions in both conifers^[32,33]^. The difference in the binding capacity between the MYB family members may be one of the possible reasons behind the inconsistency between *MYB3/MYB4* expression and lignin synthesis. A proteomic and metabolomic analysis may reveal concordance between the FTIR data and the *MYB3/MYB4* regulatory mechanism. In addition, it is the highly variable C-terminal region of different plant MYBs that contains the repressor domain, which is not characterized in conifers. For example, in *Arabidopsis*, a change (D261N) in a conserved amino acid in the GY/FDFLGL motif present in the C-terminal region of the R2R3-type MYB4 transcription repressor resulted in abolishing its repressive activity^[14]^. The N-terminal region of the different MYB members in both conifers was found to be conserved while the C-terminal region was highly variable (Supplemental Figs S1−S5). It is proposed that conifers may contain novel motifs in the C-terminal region of the MYB members that may be specific to conifer species, which needs to be functionally validated^[41]^. Furthermore, there could be conifer-specific co-repressors that interact in general with the MYB members of subgroup 4 and specifically with MYB3 to regulate the phenylpropanoid pathway similar to *Arabidopsis* where the NIGHT LIGHT-INDUCIBLE AND CLOCK-REGULATED1 (LNK1) and LNK2 act as co-repressors along with MYB3^[42]^. The LNK-MYB3 transcription complex plays a role in the repression of the *C4H* gene, one of the key genes involved in lignin biosynthesis^[42]^. Other factors contributing to the phenylpropanoid pathway regulation includes interactions between the *MYB* members that are co-expressed, their probable interactions with other transcription factors and the feedback loops. These need further investigation in conifers. Similar arguments were proposed for the detection of several grass *MYB4* homologs binding to the promoters of genes involved in the lignin biosynthesis pathway, which was not in accordance with the expression of the *MYB4* genes^[43,44]^.

The increase in lignin synthesis in response to shade in conifers^[32,33]^ is a contrasting feature compared to angiosperms, where shade causes a decrease in lignin synthesis due to which the angiosperm becomes weak and susceptible to diseases^[45,46]^. The underlying mechanism, whether and how the MYB repressors may be involved in this process in conifers needs further research.

## Conclusions

The sequence analysis suggests that although the domains of the MYB repressors from the lignin biosynthesis pathway are conserved among the angiosperms and gymnosperms in their N-terminal regions, they may possess diverse repressor domains in the C-terminal regions that have not been functionally characterized. Gymnosperms are ancient and functionally diverse compared to angiosperms in many ways. For example, comparative genome annotation studies revealed notable differences in the size of the NDH-complex gene family and the genes underlying the functional basis of response to shade suggesting specialization of the photosynthetic apparatus in Pinaceae^[47]^. Likewise, it is proposed that the lignin biosynthesis pathway in conifers may function through alternative mechanisms, unlike those observed in the angiosperms, as suggested by the study in *Ginkgo*^[30]^. Further investigation is required for functional validation of all the conifer MYB repressors discussed in this review aiming to elucidate the mechanisms underlying the repression of the conifer lignin biosynthesis pathway.

## SUPPLEMENTARY DATA

Supplementary data to this article can be found online.

## Data Availability

All data generated or analyzed during this study are included in this published article and its supplementary information files.
