# Supplementary material for: Lignin biosynthesis pathway repressors in gymnosperms: differential repressor domains as compared to angiosperms
Source: For Res (Fayettev). 2024 Sep 19;4:e031. doi: 10.48130/forres-0024-0029 (PMC11524278; doi:10.48130/forres-0024-0029)
Supplement: Supplementary file 1 — Supplementary data to this article can be found online. [file forres-0024-0029-S1.zip › 10.48130_forres-0024-0029-Suppl-FigureS2.pdf]

Figure S2 Alignment of *Arabidopsis thaliana* MYB3 (AtMYB3), *Gingko biloba* MYB (GbMYBR1) and all MYB3-like copies from *Pinus taeda* (Pt)

```
GbMYBR1              --------------------------------------------------------MGRS
Pt_AtMYB3-like2      --------------------------------------------------------MGRA
Pt_AtMYB3-like7      --------------------------------------------------------MGRA
AtMYB3               --------------------------------------------------------MGRS
Pt_AtMYB3-like5      ------------------------------MPVAGLGPGPGGGEHQPWSYRPGKYKMGRS
Pt_AtMYB3-like3      --------------------------------------------------------MGRS
Pt_AtMYB3-like6      MRKFVIKSITGRSRLPSYIYLLAITGKGRSTEKKWSLRDFIEAVGREFVGLVRSYIMGRS
Pt_AtMYB3-like1      --------------------------------------------------------MGRS
Pt_AtMYB3-like4      -------------------------------------------------------MMGRS
                                                                             ***:

GbMYBR1              P---MCSKVGLGLNKGAWTVEEDNLLIKYFQTHDEGGGWKSVPKKAGLKRCGKSCRLRWM
Pt_AtMYB3-like2      P---CCTKV--GLNKGAWSAEEDSLLGKYIQTHGE-GNWRSLPKKAGLRRCGKSCRLRWL
Pt_AtMYB3-like7      P---CCDKA--GVKRGPWTPEEDKKLLAYIEQHGL-GNWRSVPVNAGLQRCGKSCRLRWT
AtMYB3               P---CCEKA--HMNKGAWTKEEDQLLVDYIRKHGE-GCWRSLPRAAGLQRCGKSCRLRWM
Pt_AtMYB3-like5      P---CCSKE--GLNRGSWTKREDMILSEYVRIHGD-GGWRNLPEKAGLKRCGKSCRLRWL
Pt_AtMYB3-like3      R---RCSTDE-GSNRAAWTTKEDMILRDYINIHGVQGGWTSVRDQTALNRSGKSCRLRWM
Pt_AtMYB3-like6      PSHFSAKNDIAGLKRGAWTPSEDQILSDCVKIHGV-GQWKSMAKKTGLRRSAKSCRFRWL
Pt_AtMYB3-like1      PSLWSCSKQEDGINRGAWTASEDKILSEYVKTHGV-GRWRSLPIKTGLKRCAKSCRLRWL
Pt_AtMYB3-like4      PGYSSYSKHEEHLKRGAWTPSEDKILIEYIKTHGI-GQWRDLPRKAGLRRCGKSCRLRWL
                            .     :...*:  **  *   .  *.  * * .:   :.*.*..****:**

GbMYBR1              NYLRPNIKRGNISPDEEDLIIRLHGLLGNRWALIAGRIPGRTDNEIKNYWYTTLSKRVAL
Pt_AtMYB3-like2      NYLRPCIKRGNITTDEEELIIRMHALLGNRWSIIAGRVPGRTDNEIKNYWNTNLSKKLAV
Pt_AtMYB3-like7      NYLRPDIKRGKFSLQEEQTIIQLHALLGNRWSTIATHLPKRTDNEIKNYWNTHLRKRLLK
AtMYB3               NYLRPDLKRGNFTEEEDELIIKLHSLLGNKWSLIAGRLPGRTDNEIKNYWNTHIKRKLLS
Pt_AtMYB3-like5      NYLRPDIKRGNICPAEEELIIRLHRLLGNRWSLIAGRLPGRTDNEIKNYWNTHLSKKLSS
Pt_AtMYB3-like3      NYLRPNIKRGNISPEEDDLIIRLHRLLGNRWSLIAGRLPGRTDNEIKNYWNTHLRKNIGA
Pt_AtMYB3-like6      NYLRPDIKRGNISADEEELLIRLHRLLGNRWTLTTGRFPGRSDNEIKNYWNTHLRKKLQM
Pt_AtMYB3-like1      NYLRPDIKRGNISPEEEELLIRLYRLLGNRWALIAGRLPGRTDNEIKNYWNTHLSKRIQK
Pt_AtMYB3-like4      NYLRPDIKRGNISPEEDELLVRLHRLLGNRWTLIAGRLPGRTDNEIKNYWNTQLSKRVQK
                     ***** :***::   *:: ::.:: ****.*:  : ..* *:******** * : ..:

GbMYBR1              KGNEAKEHK------------TYPMKRS-----------------------RGHSACK--
Pt_AtMYB3-like2      RGIDPKTHK------KITTDGTNRVNGDG----------------------FNQRKGEK-
Pt_AtMYB3-like7      MGIDPVTHKATFDPLNLNDGQKSKEFRA-LSHKKQ----------------WENARLEA-
AtMYB3               RGIDPNSHR------LINESVVSPSS-------------------------LQNDVVET-
Pt_AtMYB3-like5      QESQDKTSK------KLSASSKSPVP-------------------------VQNRVFKA-
Pt_AtMYB3-like3      LNKRDRSEA------KMTEEDMGVSPRLDLVGANQIAVNIENNCVGRQICVWGSQRCIRG
Pt_AtMYB3-like6      G--------------------MPIDSRN-----------------------YNEKEVQD-
Pt_AtMYB3-like1      GEFKHKFQN------HFKRKHMPSDSN------------------------YGEDRVVD-
Pt_AtMYB3-like4      GEFDPEYQE------PFKERAIPAPS-------------------------IYDKGFLH-

GbMYBR1              -------------------QLIMPDSNTKMQD-----------------LLSASPTRKEL
Pt_AtMYB3-like2      -------------------IYDSPQKPRQPER-----------------NVARAAQSTGV
Pt_AtMYB3-like7      -------------------EARHRETNLNPRA-----------------SRGL-WMTGNN
AtMYB3               -------------------IHLDFSGPVKPE-----------------------PVREEI
Pt_AtMYB3-like5      -------------------TAVKITTAVRHTE------------------IIRPNACNGD
Pt_AtMYB3-like3      LRRALSRDLSARLRWLNYLRPDIKRGNISPDEEELIIRMHRLLGNRWSLIAGRLPGRTDN
Pt_AtMYB3-like6      -------------------RYDGVTQNTPLKT-----------------MPLRYWEKEEV
Pt_AtMYB3-like1      -------------------KNINAGEDTPSKA-----------------TGEISCARAVG
Pt_AtMYB3-like4      -------------------RDNDSYDILPFKT-----------------RAVRHCGRTIV

GbMYBR1              EVSTN-------------------QSLSESV---VSNTDDVRT--DSNVQSGSPGLQ---
Pt_AtMYB3-like2      VIPNVHNLKADLKAQYIARIREFKSSNTISS---SSRLNAQIEPKSRELSTEDPIFR---
Pt_AtMYB3-like7      NGSDVLNMIMLHGNNTRSTMSSA-STNNSSH---IDHLIESPTSSLSFVESSDMLPLFHH
AtMYB3               GMVN--------------------NCESSGT---TSEKDYGNE-----------------
Pt_AtMYB3-like5      GCSNFIPGEALKLCNVEN------TTKSSSCGLLVNNSSTSKQPERNAFATTDNLVQ---
Pt_AtMYB3-like3      EIKNYWNTHLSKKLTMSSQNPKF-PMPAPAD---PEQTASSESSEEDVVRSADYRVIHHS
Pt_AtMYB3-like6      DVENL-------------------DCYNYDC---PPDSHDGINPEEEGMKGGDDHVS---
Pt_AtMYB3-like1      KVDNL-------------------DKYNQDN---VPVTNEGIN-QEGGLRAADDNVV---
Pt_AtMYB3-like4      DGDNL-------------------DSYDHDC---VPDTHDGIN-QEEGLKATNHHVV---
```

```
GbMYBR1                  --EIRAS-----------------------RIFLPSFRGNQVSSHSEL--------MAPA
Pt_AtMYB3-like2          --CSSAS-----------------------EKTRETTHPDFMEP------------HPVA
Pt_AtMYB3-like7          SIPLSHQ-----------------------ESFCEYLQKKPLCEH-----------FTGI
AtMYB3                   ------------------------------EDWVLNLELSVGPSYR----------YEST
Pt_AtMYB3-like5          --TEATN-----------------------CTSSMLNFGDQQSPYSDYFEGDAANLAESL
Pt_AtMYB3-like3          GMRLAPAKIGGTSISAGVPAPVSSDEVINMKSWKQLLEDSLMSDFDDSEDDTSLMGIELS
Pt_AtMYB3-like6          --EKDAS-----------------------ETWCQLLLEDCTGYYL----------YDRL
Pt_AtMYB3-like1          --EIDTS-----------------------KSWLQLLLEDCMGEYH----------YNLL
Pt_AtMYB3-like4          --EIDTS-----------------------KSWCQLLLEDCTGDYQ----------YDRL
                                                                .
                                                      EAR – suppression domain

GbMYBR1                  N-------PMIESNIDRKLFSLVDDYLSVSTELSLGFSGMNCSVSKFS---TNSHNL---
Pt_AtMYB3-like2          S-------SDAGKQTNDSTVYCSSDSAASCSLIEHFSSEDDHHYLSMEGN-SNECYSQTL
Pt_AtMYB3-like7          D-------INNDNKTDGGSNVGDPNLLIPSSFHDPIFSDKHCNYSSTHSI-SNEISSHGD
AtMYB3                   R-------KVSVVDSAESTRRWG------SELFGAHESDAVCLCCRIGLFRNESCRN---
Pt_AtMYB3-like5          L-------FLTDLSSPDCNLLASPSDCSTDFASEDLYRGPAALPEETNGF-EDMCSDQNN
Pt_AtMYB3-like3          DDVLGSEAPLMEIQTNVSCSTSGPNEPVHSHGHLLADATSNQRSSESNVLYSQDCNVLSL
Pt_AtMYB3-like6          E-------FVQGLQTDRI-----------NLLPPSVSPSGSEILQEKQ---SSSCNL---
Pt_AtMYB3-like1          Q-------PNIMTNTKCGTDY--------DNQFPSPTLGRDILLQENG---ASSCNF---
Pt_AtMYB3-like4          E-------EISGLQPNSMTNSSNVTDDQHQHQPQSPFVSQSIILQENH---SSSCDL---
                                      .                                       .

GbMYBR1                  ------------------------------YLMGSSLSNTDHHM--------PCGDQSVC
Pt_AtMYB3-like2          AEDYGSLKPSTPHTESEPICDSRERDNDSHVQKHDQFPEYDVFSFFDV----RNAENEIC
Pt_AtMYB3-like7          LGSEISSHVDLNTDLLQQYLSEG---PSCEPAVCSELAPEDQSSIANLGSGKDYWNDILK
AtMYB3                   ------------------------------CRVSDVRTH---------------------
Pt_AtMYB3-like5          MQRVNSSSMDQELEGFYNNAQDEDWIHELDCLENSGAQP---------------LSSLLL
Pt_AtMYB3-like3          PPTDNTLDFRIQDFSLESLLLPSVYNFDEICPVQSQLNSSDHVSCFAVAAEQAMTDIYNN
Pt_AtMYB3-like6          --------------------------LEEEFLQTENFNS-------------TYF-----
Pt_AtMYB3-like1          --------------------------FDQEFPHSKEFIS-------------PYSDNCLD
Pt_AtMYB3-like4          --------------------------IQQESPHSKDFIS-------------SYFSSFLD
                                                           .

GbMYBR1                  RDQYRWMNT---------------------------------------------------
Pt_AtMYB3-like2          CSADQWVHEQEAEFMQQKDQEMAQLGSWEKQIDDQEKENFESHVNNDVTAMSWEASFWF-
Pt_AtMYB3-like7          MVNSSSSVDNIF------------------------------------------------
AtMYB3                   ------------------------------------------------------------
Pt_AtMYB3-like5          ESEDEWEERSIGKLALEDHVQI--------------------------------------
Pt_AtMYB3-like3          TLEPDWLTPVEYEKQSTQSDQMNLLGNFFLSEENWEEEVPTSLSKIQEEEYRAILVDISN
Pt_AtMYB3-like6          ------------------------------------------------------------
Pt_AtMYB3-like1          LGEPSFVIP---------------------------------------------------
Pt_AtMYB3-like4          LGEQSILLP---------------------------------------------------


GbMYBR1                  -
Pt_AtMYB3-like2          -
Pt_AtMYB3-like7          -
AtMYB3                   -
Pt_AtMYB3-like5          -
Pt_AtMYB3-like3          K
Pt_AtMYB3-like6          -
Pt_AtMYB3-like1          -
Pt_AtMYB3-like4          -
```

R2 – DNA binding domain is highlighted in yellow.
R3 – DNA binding domain is highlighted in green.
bHLH binding motif – Indicated by red box.
C1 motif – Highlighted in pink.
EAR – Suppression domain is highlighted in light blue (which comprises part of the C2 motif).
Alternative EAR domain in Pt_AtMYB4-like1, Pt_AtMYB4-like3, Pt_AtMYB4-like4 and Pa_AtMYB4-like1 are marked with box and, bold and underlined.
Zinc-finger motif – Highlighted in dark blue (C3 motif).
C4 motif – Highlighted in grey. The GY/FDFLGL motif is indicated by black box.
