# Supplementary material for: Lignin biosynthesis pathway repressors in gymnosperms: differential repressor domains as compared to angiosperms
Source: For Res (Fayettev). 2024 Sep 19;4:e031. doi: 10.48130/forres-0024-0029 (PMC11524278; doi:10.48130/forres-0024-0029)
Supplement: Supplementary file 1 — Supplementary data to this article can be found online. [file forres-0024-0029-S1.zip › 10.48130_forres-0024-0029-Suppl-FigureS3.pdf]

**Figure S3 Alignment of *Arabidopsis thaliana* MYB4 (AtMYB4), *Gingko biloba* MYB GbMYBR1) and all MYB4-like copies from *Picea abies* (Pa)**

```
GbMYBR1             MGRSPMCSKVGLGLNKGAWTVEEDNLLIKYFQTHDEGGGWKSVPKKAGLKRCGKSCRLRW
Pa_AtMYB4-like3     MGRAPCCSKV--GLNRGRWTAEEDEILTKYIQTHGEGS-WRSLPQNAGLLRCGKSCRLRW
Pa_AtMYB4-like1     MGRGPCCANG--DRNKGAWTREEDDRLIQYIQAHGEGC-WRSLPNAAGLLRCGKSCRLRW
AtMYB4              MGRSPCCEKA--HTNKGAWTKEEDERLVAYIKAHGEGC-WRSLPKAAGLLRCGKSCRLRW
Pa_AtMYB4-like2     MGRSPCCEKS--HTNKGAWTKEEDDRLIAHIRAHGEGC-WRSLPKDAGLLRCGKSCRLRW
                    ***.* * :      *.* ** ***: *  ::.:*.**  *.*:*: *** **********

GbMYBR1             MNYLRPNIKRGNISPDEEDLIIRLHGLLGNRWALIAGRIPGRTDNEIKNYWYTTLSKRVA
Pa_AtMYB4-like3     INYLRSDVKRGNICKEEEELIIRLHTLLGNRWSLIAGRMPGRTDNEIKNYWNTHLSRKLA
Pa_AtMYB4-like1     INYLRPNLKRGDFSEDEDDLILKLHALLGNRWSLIAGRLPGRTDNEIKNYWNSYLKRKLI
AtMYB4              INYLRPDLKRGNFTEEEDELIIKLHSLLGNKWSLIAGRLPGRTDNEIKNYWNTHIRRKLI
Pa_AtMYB4-like2     INYLRPDLKRGNFSEEEDELIIKLHSILGNKWSLIAARLPGRTDNEIKNYWNSHIKRKLL
                    :****.::***::  :*::**:.** :***.*:***.*:************ : :  ..:

GbMYBR1             LKGNEAKEHKTYPMKRSRGHSACKQLIMPDSNTKMQDL--LSASPTRKELEVSTNQSLS-
Pa_AtMYB4-like3     NRGIDTATHKPLGEVAEIFIQAKPSIITNVRNTSLNDIKPVRCSNIEASMVCSIDSALKK
Pa_AtMYB4-like1     SMGVDPLNHRPFQKNSRHRPSASGNVGEPGTARSRE--------IVPDFFHCPPELSTK-
AtMYB4              NRGIDPTSHRPIQES-SASQDSKPTQLEPVTSNTINIS--FTSAPKVETFHESISFPGK-
Pa_AtMYB4-like2     SRGLDPGSHRPLCFP-HHNNTTSPSRPAPDHEFPAFQN--LKTPEITDFFQQHRSES---
                      * :.   *..            :                           :    . .

GbMYBR1             -------------------ESVVSNTDDVRTDSNVQSGSPGLQEIRASRIFLPSFRGNQV
Pa_AtMYB4-like3     ISKKSSKKRSVWKEKQMPFEEQIPSGQSSSCESSCSGNLSSPDLLMAACCNPPSC--DQV
Pa_AtMYB4-like1     -------------------SDQVSDAASGLAR------KELPRFNLNLNLELSI------
AtMYB4              -------------------SEKISMLTFKEEKDECPVQEKFPDLNLELRISLPD------
Pa_AtMYB4-like2     -------------------SPIVPEASDA---------EEHPDLNLNLCISLPS------
                                       .   :.
```

EAR – suppression domain

```
GbMYBR1             SSHSELMAP---------------------ANPMIESNIDRKLFSLVDDYLSVSTELSLG
Pa_AtMYB4-like3     KKLSFEKTPCQDRSTDHVLYREIKEEDCNSDDPVNSSKLWEESFNSINILNSDMENMMFG
Pa_AtMYB4-like1     ------------------------------TRPSIHAAGKEAVVNSEQGESNLSEGKGYL
AtMYB4              ------------------------------DVDRLQGHGKSTTPRCFKCSLGMINGMECR
Pa_AtMYB4-like2     ------------------------------NSPPRREESFQRL-----------------

GbMYBR1             FSGMNCSV------SKFSTNSHNLYLMGSSLSNTDHHMPCGDQSVCRDQYRWMNT-----
Pa_AtMYB4-like3     YLETEVELFGCLADPPMPAPNADEGNMETLSSDADYNTEDELWSTFLSNSEKTTLPSPSS
Pa_AtMYB4-like1     CRHGECPP------PQSPTDG---------------------------------------
AtMYB4              CGRMRCDV------VGGSSKGSDMSNGFDFLGLAKKETTSLLGFRSLEMK----------
Pa_AtMYB4-like2     ------------------------------------------------------------

GbMYBR1             ---
Pa_AtMYB4-like3     LSA
Pa_AtMYB4-like1     ---
AtMYB4              ---
Pa_AtMYB4-like2     ---
```

**R2 – DNA binding domain is highlighted in yellow.**
**R3 – DNA binding domain is highlighted in green.**
**bHLH binding motif – Indicated by red box.**
**C1 motif – Highlighted in pink.**
**EAR – Suppression domain is highlighted in light blue (which comprises part of the C2 motif).**
**Alternative EAR domain in Pt_AtMYB4-like1, Pt_AtMYB4-like3, Pt_AtMYB4-like4 and Pa_AtMYB4-like1 are marked with box and, bold and underlined.**
**Zinc-finger motif – Highlighted in dark blue (C3 motif).**
**C4 motif – Highlighted in grey. The GY/FDFLGL motif is indicated by black box.**
