# Supplementary material for: Lignin biosynthesis pathway repressors in gymnosperms: differential repressor domains as compared to angiosperms
Source: For Res (Fayettev). 2024 Sep 19;4:e031. doi: 10.48130/forres-0024-0029 (PMC11524278; doi:10.48130/forres-0024-0029)
Supplement: Supplementary file 1 — Supplementary data to this article can be found online. [file forres-0024-0029-S1.zip › 10.48130_forres-0024-0029-Suppl-FigureS4.pdf]

Figure S4 Alignment of *Arabidopsis thaliana* MYB4 (AtMYB4), *Gingko biloba* MYB (GbMYBR1) and all MYB4-like copies from *Pinus taeda* (Pt)

```
GbMYBR1             ----------------------MGRSPMCSKVGLGLNKGAWTVEEDNLLIKYFQTHDEGG
Pt_AtMYB4-like1     ----------------------MARAPCCPIG--DRNKGAWTREEDDKLIQYIQTHGE-G
Pt_AtMYB4-like3     ----------------------MGRAPCCANG--DRSKGAWTKEEDDRLTQYIQAHGE-G
Pt_AtMYB4-like5     ----------------------MGRSPCCEKA--HTNKGAWSKEEDQVLIAYIRAHGE-G
AtMYB4              ----------------------MGRSPCCEKA--HTNKGAWTKEEDERLVAYIKAHGE-G
Pt_AtMYB4-like6     ----------------------MKR---------------------KLLIKGFDAH----
Pt_AtMYB4-like4     MSLFPLSLPSGIPVVAVFLSMKMRKYSCCEKA--HTKKGAWTQQEDARLVAYIQAHGE-G
Pt_AtMYB4-like2     ----------------------MGRTPCCEKG--HTNKGAWTKEEDDRLIAHIRAHGE-G
                                          * .                       *   : :*

GbMYBR1             GWKSVPKKAGLKRCGKSCRLRWMNYLRPNIKRGNISPDEEDLIIRLHGLLGNRWALIAGR
Pt_AtMYB4-like1     GWNSLPKAAGLLRCGKSCRLRWMNYLRPQLKRGNFSEDEDDLILKLHALLGNKWSLIAGR
Pt_AtMYB4-like3     CWRSLPKAAGLLRCGKSCRLRWINYLRPDLKRGGFSEDEDDLILKLHALLGNKWSLIAGR
Pt_AtMYB4-like5     CWRSLPKAAGLQRCGKSCRLRWINYLRPDLKRGNFSSEEDDLIIKFHSMLGNKWSLIASR
AtMYB4              CWRSLPKAAGLLRCGKSCRLRWINYLRPDLKRGNFTEEEDELIIKLHSLLGNKWSLIAGR
Pt_AtMYB4-like6     -----------------------------LKRGNFSEEEDELIIRLHSLLGNKWSLIAGR
Pt_AtMYB4-like4     GWRYLPKAAGLLRCGKSCRLRWINYLRPDLKRGNFSEEEDELIIKLHSLLGNKWSLIAAR
Pt_AtMYB4-like2     RWRSLPKAAGLMRCGKSCRLRWINYLRPDLKRGNFSEEEDEFIIKLHSIIGNKWSVIAAR
                                                 :***.:: :*:::*:.:*.::**.*::**.*

GbMYBR1             IPGRTDNEIKNYWYTTLSKRVALKGNEAKEHKTYPMKRSRGHSACKQLIMPDSNTKMQDL
Pt_AtMYB4-like1     LPGRTDNEIKNYWNSHLKRKLISMGVDPLTHR--PFQKICRH------------------
Pt_AtMYB4-like3     LPGRTDNEIKNYWNSHLKRKLISMGVDPLTRR--PFQRNSRH------------------
Pt_AtMYB4-like5     LPGRTDNEIKNYWNTHIKRKLLEKGIDPNTHL--PILPDHGS------------------
AtMYB4              LPGRTDNEIKNYWNTHIRRKLINRGIDPTSHR--PIQESSAS------------------
Pt_AtMYB4-like6     LPGRTGNEIKNHWSIHMKRKLSSRGLHPQTHR--SLRPPHNS------------------
Pt_AtMYB4-like4     MAGRTDNEIKNYWNTHIKRKLLSRGLDPQTHR--SLCPPHNS------------------
Pt_AtMYB4-like2     LPGRTDNEIKNYWNSHIKRKLLSRGIDPRSHR--PLCPPSNT------------------
                    :.***.*****:*   : ..:   *  .  .   .:

GbMYBR1             LSASPTRKELEVSTNQSLSESVVSNTDDVRTDSNVQSGSPGLQEIRASRIFLPSFRGNQV
Pt_AtMYB4-like1     --------------DQPLSE-----NVSE----------PEIASSGKF---VQDFFHC-P
Pt_AtMYB4-like3     --------------DLSLPE-----KVEV----------PEIARPREI---VENFFHS-P
Pt_AtMYB4-like5     --------------FGSSNE-----NHVF----------SKRSRCKPSEV-LTDFFQQ--
AtMYB4              --------------QDSKPTQLEPVTSNT----------INISFTSAPK--VETFHESIS
Pt_AtMYB4-like6     --------------STTLNSPPPP-DHQI----------LAFESRRRAE--IADFFQL--
Pt_AtMYB4-like4     --------------NSTLSSPSVP-EHEI----------SPLQNPRTAD--IADFFQQ--
Pt_AtMYB4-like2     -------------TSLSWSAP----DREF----------PAIQNLRTPE--ITDFFQH--
                                    .                                  :  *

GbMYBR1             SSHSELMAPANPMIESNIDRKLFSLVDDYLSVSTELSLGFSGMNCSVSKFS---------
Pt_AtMYB4-like1     SELSVESAQLSNAAGGSLARDGQ---PD---LNLNLDLGLTVTCPSIHSEG---------
Pt_AtMYB4-like3     SELCGDSEQVSDAASESLARDE----PSTLNLNLELSINWASMHVAVKEEP---------
Pt_AtMYB4-like5     -QTPGGTSSVLKVIAKNQDHDYD---KD---VNLDLSITLPSLCSSQGSSSSSRELSSGI
AtMYB4              FPGKSEKISMLTFKEEKDECPVQEKFPD---LNLELRISLPDDVDRLQGHG-----KSTT
Pt_AtMYB4-like6     --EGLKSSPIESAASTDEEHPC----PD---INLDLCMGLSSNSAPA-------------
Pt_AtMYB4-like4     --DRSERSPVENAACKEEEH------PD---LNLDLCISLPSNSTLAAN-----------
Pt_AtMYB4-like2     --NRSESSPILPAAPDAEED------PD---LNLNLCISLPSNSSPAANTA------QSV
                                               .   :. :* :  .
                                         EAR – suppression domain

GbMYBR1             -TNSHNLYLMGSSLSNTDHHMPCGDQSVCRDQYRWMNT----------------------
Pt_AtMYB4-like1     -KQNVVNSEQGENDLGEGMGYLAYHSGECAPPMLLLR-----------------------
Pt_AtMYB4-like3     ILKSKPGEEEGAGGRFDEKGYLFRYEECPVGKSLLR------------------------
Pt_AtMYB4-like5     RLSNDCTQQDHKEKICCHIGLRTVGESCCSG--CWD------------------------
AtMYB4              PRCFKCSLGMINGMECRCGRMRCDVVGGSSKGSDMSNGFDFLGLAKKETTSLLGFRSLEM
Pt_AtMYB4-like6     ------------------------------------------------------------
Pt_AtMYB4-like4     -RAMDANSNSGLGVWCH-TGPQINSDPRCENRYCERNSFTELDSSSGSIHGILDLQLSAL
Pt_AtMYB4-like2     RKTVDSNLNSGSSEVCYPVGLQINYGKYCDSGYCEQNASCFSQFRLVL------------


GbMYBR1             ---
Pt_AtMYB4-like1     ---
Pt_AtMYB4-like3     ---
Pt_AtMYB4-like5     ---
AtMYB4              K--
Pt_AtMYB4-like6     ---
Pt_AtMYB4-like4     GVQ
Pt_AtMYB4-like2     ---
```

R2 – DNA binding domain is highlighted in yellow.
R3 – DNA binding domain is highlighted in green.
bHLH binding motif – Indicated by red box.
C1 motif – Highlighted in pink.
EAR – Suppression domain is highlighted in light blue (which comprises part of the C2 motif).
Alternative EAR domain in Pt_AtMYB4-like1, Pt_AtMYB4-like3, Pt_AtMYB4-like4 and Pa_AtMYB4-like1 are marked with box and, bold and underlined.
Zinc-finger motif – Highlighted in dark blue (C3 motif).
C4 motif – Highlighted in grey. The GY/FDFLGL motif is indicated by black box.
