# Supplementary material for: Lignin biosynthesis pathway repressors in gymnosperms: differential repressor domains as compared to angiosperms
Source: For Res (Fayettev). 2024 Sep 19;4:e031. doi: 10.48130/forres-0024-0029 (PMC11524278; doi:10.48130/forres-0024-0029)
Supplement: Supplementary file 1 — Supplementary data to this article can be found online. [file forres-0024-0029-S1.zip › 10.48130_forres-0024-0029-Suppl-FigureS5.pdf]

Figure S5 Alignment of the lignin repressor MYB members from gymnosperm and angiosperm species: MYB3-like and MYB4-like copies from *Picea abies* (Pa) and *Pinus taeda* (Pt) along with GbMYBR1 from *Ginkgo biloba* (Gb); CfMYB5 from *Cryptomeria fortune* (Cf); MYB3, MYB4, MYB7 and MYB32 from *Arabidopsis thaliana* (At); MYB156 and MYB221 from *Populus trichocarpa* (Potri); EgMYB1 from *Eucalyptus gunnii* (Eg); ZmMYB31 and ZmMYB42 from *Zea mays* (Zm) and PvMYB4 from *Panicum virgatum* (Pv). Alternative EAR domain in Pt_AtMYB4-like1, Pt_AtMYB4-like3, Pt_AtMYB4-like4 and Pa_AtMYB4-like1 are marked with box and, bold and underlined.

```
CfMYB5               --------------------------------------------------------MGRS
GbMYBR1              --------------------------------------------------------MGRS
Pa_AtMYB3-like1      --------------------------------------------------------MGRA
Pt_AtMYB3-like2      --------------------------------------------------------MGRA
Pa_AtMYB4-like3      --------------------------------------------------------MGRA
Pt_AtMYB4-like1      --------------------------------------------------------MARA
Pt_AtMYB4-like3      --------------------------------------------------------MGRA
Pa_AtMYB4-like1      --------------------------------------------------------MGRG
AtMYB3               --------------------------------------------------------MGRS
Pt_AtMYB4-like6      --------------------------------------------------------MKR-
AtMYB32              --------------------------------------------------------MGRS
AtMYB7               --------------------------------------------------------MGRS
AtMYB4               --------------------------------------------------------MGRS
Pt_AtMYB4-like5      --------------------------------------------------------MGRS
EgMYB1               --------------------------------------------------------MGRS
Potri_MYB221         --------------------------------------------------------MGRS
Potri_MYB156         --------------------------------------------------------MGRS
Pt_AtMYB4-like4      ----------------------------------MSLFPLSLPSGIPVVAVFLSMKMRKY
Pa_AtMYB4-like2      --------------------------------------------------------MGRS
Pt_AtMYB4-like2      --------------------------------------------------------MGRT
ZmMYB31              --------------------------------------------------------MGRS
PvMYB4               --------------------------------------------------------MGRS
ZmMYB42              --------------------------------------------------------MGRS
Pt_AtMYB3-like6      MRKFVIKSITGRSRLPSYIYLLAITGKGRSTEKKWSLRDFIEAVGREFVGLVRSYIMGRS
Pt_AtMYB3-like4      -------------------------------------------------------MMGRS
Pa_AtMYB3-like4      --------------------------------------------------------MGRS
Pt_AtMYB3-like1      --------------------------------------------------------MGRS
Pt_AtMYB3-like3      --------------------------------------------------------MGRS
Pa_AtMYB3-like3      --------------------------------------------------------MGRP
Pt_AtMYB3-like5      ------------------------------MPVAGLGPGPGGGEHQPWSYRPGKYKMGRS
Pa_AtMYB3-like2      --------------------------------------------------------MGRS
Pa_AtMYB3-like5      --------------------------------------------------------MGRS
                                                                             * .
```

R2 Domain

```
CfMYB5               AECE-----KVHTNKGPWSPEEDALLQCYVQKYG-ADNWSLISKGAGLRLCGKSCRLRWC
GbMYBR1              PMCS---KVGLGLNKGAWTVEEDNLLIKYFQTHDEGGGWKSVPKKAGLKRCGKSCRLRWM
Pa_AtMYB3-like1      PCCA-----KVGLNKGAWSAEEDSLLGKYIQTHG-EGNWRSLPKKAGLRRCGKSCRLRWL
Pt_AtMYB3-like2      PCCT-----KVGLNKGAWSAEEDSLLGKYIQTHG-EGNWRSLPKKAGLRRCGKSCRLRWL
Pa_AtMYB4-like3      PCCS-----KVGLNRGRWTAEEDEILTKYIQTHG-EGSWRSLPQNAGLLRCGKSCRLRWI
Pt_AtMYB4-like1      PCCP-----IGDRNKGAWTREEDDKLIQYIQTHG-EGGWNSLPKAAGLLRCGKSCRLRWM
Pt_AtMYB4-like3      PCCA-----NGDRSKGAWTKEEDDRLTQYIQAHG-EGCWRSLPKAAGLLRCGKSCRLRWI
Pa_AtMYB4-like1      PCCA-----NGDRNKGAWTREEDDRLIQYIQAHG-EGCWRSLPNAAGLLRCGKSCRLRWI
AtMYB3               PCCE-----KAHMNKGAWTKEEDQLLVDYIRKHG-EGCWRSLPRAAGLQRCGKSCRLRWM
Pt_AtMYB4-like6      -----------------------KLLIKGFDAH---------------------------
AtMYB32              PCCE-----KDHTNKGAWTKEEDDKLISYIKAHG-EGCWRSLPRSAGLQRCGKSCRLRWI
AtMYB7               PCCE-----KEHMNKGAWTKEEDERLVSYIKSHG-EGCWRSLPRAAGLLRCGKSCRLRWI
AtMYB4               PCCE-----KAHTNKGAWTKEEDERLVAYIKAHG-EGCWRSLPKAAGLLRCGKSCRLRWI
Pt_AtMYB4-like5      PCCE-----KAHTNKGAWSKEEDQVLIAYIRAHG-EGCWRSLPKAAGLQRCGKSCRLRWI
EgMYB1               PCCE-----KAHTNKGAWTKEEDDKLIAYIRAHG-EGCWRSLPKAAGLLRCGKSCRLRWI
Potri_MYB221         PCCE-----KAHTNKGAWTKEEDDRLIAYIRTHG-EGCWRSLPKAAGLLRCGKSCRLRWI
Potri_MYB156         PCCE-----KAHTNKGAWTKEEDDRLVAYIRAHG-EGCWRSLPKAAGLLRCGKSCRLRWI
Pt_AtMYB4-like4      SCCE-----KAHTKKGAWTQQEDARLVAYIQAHG-EGGWRYLPKAAGLLRCGKSCRLRWI
Pa_AtMYB4-like2      PCCE-----KSHTNKGAWTKEEDDRLIAHIRAHG-EGCWRSLPKDAGLLRCGKSCRLRWI
Pt_AtMYB4-like2      PCCE-----KGHTNKGAWTKEEDDRLIAHIRAHG-EGRWRSLPKAAGLMRCGKSCRLRWI
ZmMYB31              PCCE-----KAHTNKGAWTKEEDERLVAHIRAHG-EGCWRSLPKAAGLLRCGKSCRLRWI
PvMYB4               PCCE-----KAHTNKGAWTKEEDDRLVAYIRAHG-EGCWRSLPKAAGLLRCGKSCRLRWI
ZmMYB42              PCCE-----KAHTNRGAWTKEEDERLVAYVRAHG-EGCWRSLPRAAGLLRCGKSCRLRWI
Pt_AtMYB3-like6      PSHFSAKNDIAGLKRGAWTPSEDQILSDCVKIHG-VGQWKSMAKKTGLRRSAKSCRFRWL
Pt_AtMYB3-like4      PGYSSYSKHEEHLKRGAWTPSEDKILIEYIKTHG-IGQWRDLPRKAGLRRCGKSCRLRWL
Pa_AtMYB3-like4      PSCSSYSKHEDGLNRGAWTAGEDKILSEYVKTHG-VGRWRSLPKKAGLRRCGKSCRLRWL
Pt_AtMYB3-like1      PSLWSCSKQEDGINRGAWTASEDKILSEYVKTHG-VGRWRSLPIKTGLKRCAKSCRLRWL
Pt_AtMYB3-like3      RRCS----TDEGSNRAAWTTKEDMILRDYINIHGVQGGWTSVRDQTALNRSGKSCRLRWM
Pa_AtMYB3-like3      QSCS-----GLKLRKGAWTSEEDLLLKKCIEVHG-EGSWPLLPQKTGLQRSGKSCRLRWL
Pt_AtMYB3-like5      PCCS-----KEGLNRGSWTKREDMILSEYVRIHG-DGGWRNLPEKAGLKRCGKSCRLRWL
Pa_AtMYB3-like2      PCCS-----KEGLNRGAWTKTEDIILCEYIRIHG-DGGWRTLPKKAGLKRCGKSCRLRWL
Pa_AtMYB3-like5      -CCS-----KEGLNRGAWSRKEDMILSEYIRIHG-DGGWTNLPQRAGLKRCGKSCRLRWT
                                              *   .  :
```

```
                     R2 Domain               R3 Domain
                                       bHLH Domain

CfMYB5               NQLRPQVKHGPFTPAEEALIVELHAKLGNKWATIARYLPGRTDNEIKNHWNSTLRRRC--
GbMYBR1              NYLRPNIKRGNISPDEEDLIIRLHGLLGNRWALIAGRIPGRTDNEIKNYWYTTLSKRV--
Pa_AtMYB3-like1      NYLRPCIKRGNITADEEELIIRMHALLGNRWSIIAGRVPGRTDNEIKNYWNTNLSKKL--
Pt_AtMYB3-like2      NYLRPCIKRGNITTDEEELIIRMHALLGNRWSIIAGRVPGRTDNEIKNYWNTNLSKKL--
Pa_AtMYB4-like3      NYLRSDVKRGNICKEEEELIIRLHTLLGNRWSLIAGRMPGRTDNEIKNYWNTHLSRKL--
Pt_AtMYB4-like1      NYLRPQLKRGNFSEDEDDLILKLHALLGNKWSLIAGRLPGRTDNEIKNYWNSHLKRKL--
Pt_AtMYB4-like3      NYLRPDLKRGGFSEDEDDLILKLHALLGNKWSLIAGRLPGRTDNEIKNYWNSHLKRKL--
Pa_AtMYB4-like1      NYLRPNLKRGDFSEDEDDLILKLHALLGNRWSLIAGRLPGRTDNEIKNYWNSYLKRKL--
AtMYB3               NYLRPDLKRGNFTEEEDELIIKLHSLLGNKWSLIAGRLPGRTDNEIKNYWNTHIKRKL--
Pt_AtMYB4-like6      ------LKRGNFSEEEDELIIRLHSLLGNKWSLIAGRLPGRTGNEIKNHWSIHMKRKL--
AtMYB32              NYLRPDLKRGNFTLEEDDLIIKLHSLLGNKWSLIATRLPGRTDNEIKNYWNTHVKRKL--
AtMYB7               NYLRPDLKRGNFTHDEDELIIKLHSLLGNKWSLIAARLPGRTDNEIKNYWNTHIKRKL--
AtMYB4               NYLRPDLKRGNFTEEEDELIIKLHSLLGNKWSLIAGRLPGRTDNEIKNYWNTHIRRKL--
Pt_AtMYB4-like5      NYLRPDLKRGNFSSEEDDLIIKFHSMLGNKWSLIASRLPGRTDNEIKNYWNTHIKRKL--
EgMYB1               NYLRPDLKRGNFTEEEDEIIIKLHSLLGNKWSLIAGRLPGRTDNEIKNYWNTHIRRKL--
Potri_MYB221         NYLRPDLKRGNFTEEEDELIIKLHSLLGNKWSLIAGRLPGRTDNEIKNYWNTHIRRKL--
Potri_MYB156         NYLRPDLKRGNFTEAEDELIIKLHSLLGNKWSLIAGRLPGRTDNEIKNYWNTHIRRKL--
Pt_AtMYB4-like4      NYLRPDLKRGNFSEEEDELIIKLHSLLGNKWSLIAARMAGRTDNEIKNYWNTHIKRKL--
Pa_AtMYB4-like2      NYLRPDLKRGNFSEEEDELIIKLHSILGNKWSLIAARLPGRTDNEIKNYWNSHIKRKL--
Pt_AtMYB4-like2      NYLRPDLKRGNFSEEEDEFIIKLHSIIGNKWSVIAARLPGRTDNEIKNYWNSHIKRKL--
ZmMYB31              NYLRPDLKRGNFTEEEDELIVKLHSVLGNKWSLIAGRLPGRTDNEIKNYWNTHIRRKL--
PvMYB4               NYLRPDLKRGNFTADEDDLIVKLHSLLGNKWSLIAARLPGRTDNEIKNYWNTHIKRKL--
ZmMYB42              NYLRPDLKRGNFTADEDDLIVKLHSLLGNKWSLIAARLPGRTDNEIKNYWNTHIRRKL--
Pt_AtMYB3-like6      NYLRPDIKRGNISADEEELLIRLHRLLGNRWTLTTGRFPGRSDNEIKNYWNTHLRKKL--
Pt_AtMYB3-like4      NYLRPDIKRGNISPEEDELLVRLHRLLGNRWTLIAGRLPGRTDNEIKNYWNTQLSKRV--
Pa_AtMYB3-like4      NYLRPDIKRGNISPEEEELIIRLYRLLGNRWALISGRLPGRTDNEIKNYWNTHLSKKV--
Pt_AtMYB3-like1      NYLRPDIKRGNISPEEEELLIRLYRLLGNRWALIAGRLPGRTDNEIKNYWNTHLSKRI--
Pt_AtMYB3-like3      NYLRPNIKRGNISPEEDDLIIRLHRLLGNRWSLIAGRLPGRTDNEIKNYWNTHLRKNIGA
Pa_AtMYB3-like3      NYLSPNVKRGNISEDEELLIIRMHRLLGNRWALIAGRIPGRSDNEIKNYWNTHLSKKV--
Pt_AtMYB3-like5      NYLRPDIKRGNICPAEEELIIRLHRLLGNRWSLIAGRLPGRTDNEIKNYWNTHLSKKL--
Pa_AtMYB3-like2      NYLRPDIKRGGISPDEEELIIRLHRLLGNRWSLIAGRLPGRTDNEIKNYWNTCLSKKL--
Pa_AtMYB3-like5      NYLRPDIKLGNISPDEDELIIRMHRLLGNRWSLIAGRLPGRTDNEIKNYWNTRLSKKL--
                           :* * :   *: ::: ::  :**.*:  :  ..**:.*****:*   : ..


CfMYB5               -----Q----------DSNP----------------------------------------
GbMYBR1              ---ALK----------GNEA----------------------------------------
Pa_AtMYB3-like1      ---AVR----------GIDP----------------------------------------
Pt_AtMYB3-like2      ---AVR----------GIDP----------------------------------------
Pa_AtMYB4-like3      ---ANR----------GIDT----------------------------------------
Pt_AtMYB4-like1      ---ISM----------GVDP----------------------------------------
Pt_AtMYB4-like3      ---ISM----------GVDP----------------------------------------
Pa_AtMYB4-like1      ---ISM----------GVDP----------------------------------------
AtMYB3               ---LSR----------GIDP----------------------------------------
Pt_AtMYB4-like6      ---SSR----------GLHP----------------------------------------
AtMYB32              ---LRK----------GIDP----------------------------------------
AtMYB7               ---LSK----------GIDP----------------------------------------
AtMYB4               ---INR----------GIDP----------------------------------------
Pt_AtMYB4-like5      ---LEK----------GIDP----------------------------------------
EgMYB1               ---LNR----------GIDP----------------------------------------
Potri_MYB221         ---LNR----------GIDP----------------------------------------
Potri_MYB156         ---LNR----------GIDP----------------------------------------
Pt_AtMYB4-like4      ---LSR----------GLDP----------------------------------------
Pa_AtMYB4-like2      ---LSR----------GLDP----------------------------------------
Pt_AtMYB4-like2      ---LSR----------GIDP----------------------------------------
ZmMYB31              ---LSR----------GIDP----------------------------------------
PvMYB4               ---LSR----------GIDP----------------------------------------
ZmMYB42              ---LGS----------GIDP----------------------------------------
Pt_AtMYB3-like6      ----QM----------GM-P----------------------------------------
Pt_AtMYB3-like4      ---QKG----------EFDP----------------------------------------
Pa_AtMYB3-like4      ---RMG----------ESDP----------------------------------------
Pt_AtMYB3-like1      ---QKG----------EFKH----------------------------------------
Pt_AtMYB3-like3      LNKRDRSEAKMTEEDMGVSPRLDLVGANQIAVNIENNCVGRQICVWGSQRCIRGLRRALS
Pa_AtMYB3-like3      --NRSR----------SDDF----------------------------------------
Pt_AtMYB3-like5      ---SSQ----------ESQD----------------------------------------
Pa_AtMYB3-like2      LLSMNK----------SQSK----------------------------------------
Pa_AtMYB3-like5      ---L------------SIDD----------------------------------------
```

```
CfMYB5              -------------------------------GLAAAVTH---------NED---------
GbMYBR1             -------------------------------KEHKTYPM---------KRS---------
Pa_AtMYB3-like1     -------------------------------KTHKKVTT---------DSINRASDRFNQ
Pt_AtMYB3-like2     -------------------------------KTHKKITT---------DGTNRVNGDGFN
Pa_AtMYB4-like3     -------------------------------ATHKPLGE---------VAEIFIQAKP--
Pt_AtMYB4-like1     -------------------------------LTHRPFQK---------ICR---------
Pt_AtMYB4-like3     -------------------------------LTRRPFQR---------NSR---------
Pa_AtMYB4-like1     -------------------------------LNHRPFQK---------NSR---------
AtMYB3              -------------------------------NSHRLINE---------SVVSPSSLQND-
Pt_AtMYB4-like6     -------------------------------QTHRSLRP---------PH----------
AtMYB32             -------------------------------ATHRPINE---------TKTSQDS-----
AtMYB7              -------------------------------ATHRGINE---------AKI---------
AtMYB4              -------------------------------TSHRPIQE---------SSASQD------
Pt_AtMYB4-like5     -------------------------------NTHLPILP---------DHGS--------
EgMYB1              -------------------------------ATHRLINE---------PAQD--------
Potri_MYB221        -------------------------------ATHRPLNE---------PA----------
Potri_MYB156        -------------------------------ATHRPLNE---------PAV---------
Pt_AtMYB4-like4     -------------------------------QTHRSLCP---------PH----------
Pa_AtMYB4-like2     -------------------------------GSHRPLCF---------PH----------
Pt_AtMYB4-like2     -------------------------------RSHRPLCP---------P-----------
ZmMYB31             -------------------------------VTHRPVTE---------H-----------
PvMYB4              -------------------------------VTHRPIAD---------------------
ZmMYB42             -------------------------------VTHRRVAG---------------------
Pt_AtMYB3-like6     -------------------------------IDSRNYNE---------KEVQDRY-----
Pt_AtMYB3-like4     -------------------------------EYQEPFKE---------RAIPAPSIYDKG
Pa_AtMYB3-like4     -------------------------------RFQNLCKR---------RDVPPYPNYD--
Pt_AtMYB3-like1     -------------------------------KFQNHFKR---------KHMPSDSNYG--
Pt_AtMYB3-like3     RDLSARLRWLNYLRPDIKRGNISPDEEELIIRMHRLLGNRWSLIAGRLPGRTDNEIKNYW
Pa_AtMYB3-like3     -------------------------------NCNKE------------------------
Pt_AtMYB3-like5     -------------------------------KTSKKLSA---------SSKSPVP-----
Pa_AtMYB3-like2     -------------------------------VTKNVKST---------SK----------
Pa_AtMYB3-like5     -------------------------------SQSKSTAR---------------------


CfMYB5              --------NSSSLKRSSCSNDDGS-------------STLQDENCGFEEA----------
GbMYBR1             --------RGHSACKQLIMPDSNT----KMQDLLSASPTRKELEVSTNQS----------
Pa_AtMYB3-like1     RKGGKLY-DCSQRSQRLERNVVRAGQSTGLVIPNVHNLKADLKGQYIAGPREIQSSNSIR
Pt_AtMYB3-like2     QRKGEKIYDSPQKPRQPERNVARAAQSTGVVIPNVHNLKADLKAQYIARIREFKSSNTIS
Pa_AtMYB4-like3     --------SIITNVRNTSLNDIKP-------------VRCSNIEASMVCSIDSALKKISK
Pt_AtMYB4-like1     --------HDQPLSENVSEPEIAS--------------SGKFVQDFFHC-----------
Pt_AtMYB4-like3     --------HDLSLPEKVEVPEIAR--------------PREIVENFFHSP----------
Pa_AtMYB4-like1     --------HRPSASGNVGEPGTAR--------------SREIVPDFFHCP----------
AtMYB3              --------VVETIHLDFSGPVKPE-------------PVREEIGMVNNCE----------
Pt_AtMYB4-like6     --------NSSTTLNSPPPPDHQI--------LAFESRRRAEIADFFQLEGLKS------
AtMYB32             --------SDSSKTEDPLVKILSFG------------PQLEKIANFGDER----------
AtMYB7              --------SDLKKTKDQIVKDVSF-------------VTKFEETDKSGDQ----------
AtMYB4              --------SKPTQLEPVTSNTINISF-----------TSAPKVETFHESI----------
Pt_AtMYB4-like5     --------FGSSNENHVFSKRSRC-------------KPSEVLTDFFQQQ----------
EgMYB1              --------HHDEPTISFAANSKEI----------------KEMKN---------------
Potri_MYB221        --------QEASTTISFSTTTS---------------VKEESLSSVKEES----------
Potri_MYB156        --------QEATTTISFTTTTTSV-------------LEEESLGSIIKEE----------
Pt_AtMYB4-like4     --------NSNSTLSSPSVPEHEI--------SPLQNPRTADIADFFQQD----------
Pa_AtMYB4-like2     --------HNNTTSPSRPAPDHEF--------PAFQNLKTPEITDFFQQH----------
Pt_AtMYB4-like2     ---------SNTTSLSWSAPDREF--------PAIQNLRTPEITDFFQHN----------
ZmMYB31             --------HASNITISFETEVAAA-------------ARDDKKGAVFRLE----------
PvMYB4              --------AARNVTISFQPDA----------------PSQQQLSDDAEAP----------
ZmMYB42             --------GAATTISFQPSPNTAV-------------AAAAE------------------
Pt_AtMYB3-like6     --------DGVTQNTPLKTMPLRYWEKEE--------VDVENLDCYNYDC----------
Pt_AtMYB3-like4     FLHRD---NDSYDILPFKTRAVRH--------CGRTIVDGDNLDSYDHDC----------
Pa_AtMYB3-like4     --------EDIYASQNAPVKTTGVRYGRAV-------AELENFDSYNPDH----------
Pt_AtMYB3-like1     --------EDRVVDKNINAGEDTPSKATGEISCARAVGKVDNLDKYNQDN----------
Pt_AtMYB3-like3     NTHLSKKLTMSSQNPKFPMPAPADPEQTASSESSEEDVVRSADYRVIHHSGMRLAPAKIG
Pa_AtMYB3-like3     -----------NVVQTLTSEEGKG-------------GQEIGAENYSSHS----------
Pt_AtMYB3-like5     --------VQNRVFKATAVKITTA-------------VRHTEIIRPNACN----------
Pa_AtMYB3-like2     --------SSPVQNRVFKTTPVKV-------------TTAVRLSETIRPK----------
Pa_AtMYB3-like5     --------NLETSSKSPPPPPNHV--FKTTPIKITTAVKFSETVGPKRCN----------
```

```
CfMYB5             ---------DS----KR----------------------------------------LKS
GbMYBR1            ---------------------------------------------------------LSE
Pa_AtMYB3-like1    SSSPINTPIQPKSNDFMQPQPVVSSDAGKQ---------------------------TDD
Pt_AtMYB3-like2    SSSRLNAQIEPKSRELSTEDPIFRCSSASEKTRETTHPDFMEPHPVASSDAGKQ---TND
Pa_AtMYB4-like3    KSSKKRSVWKEK---QM----------------------------------------PFE
Pt_AtMYB4-like1    ---------------------------------------------------------PSE
Pt_AtMYB4-like3    ---------SEL---CG----------------------------------------DSE
Pa_AtMYB4-like1    ---------------PE----------------------------------------LST
AtMYB3             ---------SSG---------------------------------------------TTS
Pt_AtMYB4-like6    ---------------------------------------------------------SPI
AtMYB32            ---------------------------------------------------------IQK
AtMYB7             ---------KQ----NK----------------------------------------YIR
AtMYB4             ---------SFPGKSEK----------------------------------------ISM
Pt_AtMYB4-like5    ---------TPG---GT----------------------------------------SSV
EgMYB1             ---------------------------------------------------------NAE
Potri_MYB221       ---------NKE---KI----------------------------------------ISA
Potri_MYB156       ---------NKE---KI----------------------------------------ISA
Pt_AtMYB4-like4    ---------RS----ER----------------------------------------SPV
Pa_AtMYB4-like2    ---------------------------------------------------------RSE
Pt_AtMYB4-like2    ---------RS----ES----------------------------------------SPI
ZmMYB31            ---------EEEERNKATMVVGRD---------------------------------RQS
PvMYB4             ---------------------------------------------------------PPP
ZmMYB42            ---------------------------------------------------------TAA
Pt_AtMYB3-like6    ---------------------------------------------------------PPD
Pt_AtMYB3-like4    ---------------------------------------------------------VPD
Pa_AtMYB3-like4    ---------------------------------------------------------VPD
Pt_AtMYB3-like1    ---------------------------------------------------------VPV
Pt_AtMYB3-like3    GTSISAGVPAPVSSDEVINMKSWKQLLEDSLMSDFDDSEDDTSLMGIELSDDVLGSEAPL
Pa_AtMYB3-like3    ---------------------------------------------------------QPK
Pt_AtMYB3-like5    ---------GDGCSNFI----------------------------------------PGE
Pa_AtMYB3-like2    ---------------------------------------------------------GSM
Pa_AtMYB3-like5    ---------GYGRSNCS----------------------------------------SAE
```

EAR
suppression domain

```
CfMYB5             HGNDCLEES---------KQAPQVVYKPVPVASC------------GFLKPPQQEAT---
GbMYBR1            SVVSNTDDVRTDSNVQSGSPGLQEIRASRIFLPS--------FRGNQVSSHSELMAPANP
Pa_AtMYB3-like1    STVYCSSDSAASCALIDHLSSEDDDQYLSLEGNSNECYSHTVAEESGTLKSSNPQTHSEA
Pt_AtMYB3-like2    STVYCSSDSAASCSLIEHFSSEDDHHYLSMEGNSNECYSQTLAEDYGSLKPSTPHTESEP
Pa_AtMYB4-like3    EQIPSGQSSSCESSCSGNLSSPDLLMAACCNPPSCDQVKKLSFEKTPCQDRSTDHVLYRE
Pt_AtMYB4-like1    LSVESAQLSNAAGGSLARDGQPDLNLNLDLGLTV---------------TCPSIHSEG--
Pt_AtMYB4-like3    QVSDAASES----LARD-EPS-TLNLNLELSINW--A-------------SMHVAVKEEP
Pa_AtMYB4-like1    KSDQVSDAASGLARKELPRFNLNLNLELSITRPS--------------IHAAGKEAVVNS
AtMYB3             EKDYGNEED------------WVLNLELSVGPSY---------RYESTRKVSVVDSAEST
Pt_AtMYB4-like6    ESAASTDEE---------HPCPDINLDLCMGLSS--------------NSAPA-------
AtMYB32            RVEYSVVEE----------RCLDLNLELRISPPW--------------QDKLHDE-----
AtMYB7             NGLVCKEER----VVVEEKIGPDLNLELRISPPW--------------------------
AtMYB4             LTFKEEKDE-----CPVQEKFPDLNLELRISLPD----------------DVDR------
Pt_AtMYB4-like5    LKVIAKNQD--------HDYDKDVNLDLSITLPS--------LCSSQGSSSSSRELSSGI
EgMYB1             LNFMCNLEESADVASSARERCPDLNLELGISPPS------------HQLHQPEP------
Potri_MYB221       AAFICKEEK-----TPVQERCPDLNLELRISLPC--------------QNQPDRH-----
Potri_MYB156       TAFVCKEEK-----TQVQERCPDLNLELGISLPS--------------QNQPDHH-----
Pt_AtMYB4-like4    ENAACKEEE-----------HPDLNLDLCISLPS--------------NSTLAAN-----
Pa_AtMYB4-like2    SSPIVPEAS-------DAEEHPDLNLNLCISLPS--------------NSPPRRE-----
Pt_AtMYB4-like2    LPAAPDAEE-----------DPDLNLNLCISLPS--------------NSSPAANTAQSV
ZmMYB31            QSQSHSHPAGEWGQGKRPLKCPDLNLDLCISPPC--------------QEEEEMEEAAMR
PvMYB4             PPPQQQQQQ-----LKPPPRCPDLNLDLCISPPC-------------HKEEEDQELIKPA
ZmMYB42            QAPIKAEET----AAVKAPRCPDLNLDLCISPPC------QHEDDGEEEEEELDLIKPAV
Pt_AtMYB3-like6    SHDGINPEE------EGMKGGDDHVSEKDASETWCQLLLEDCTGYYLYDRLEFVQGL---
Pt_AtMYB3-like4    THDGINQEE-------GLKATNHHVVEIDTSKSWCQLLLEDCTGDYQYDRLEEISGLQPN
Pa_AtMYB3-like4    THDGSTQEG--------LKAADDHVVEIDTSKSWSQLLLEHCMGDYQYDRLQPNASS---
Pt_AtMYB3-like1    TNEGINQEG-------GLRAADDNVVEIDTSKSWLQLLLEDCMGEYHYNLLQPNIMTNTK
Pt_AtMYB3-like3    MEIQTNVSCSTSGPNEPVHSHGHLLADATSNQRSSESNVLYSQDCNVLSLPPTDNTLDFR
Pa_AtMYB3-like3    SRISINTDH---------NQHADMLEEPSTGLTK----------CQSYRNTPTEQTDL--
Pt_AtMYB3-like5    ALKLCNVEN-----TTKSSSCGLLVNNSSTSKQPERNAFATTDNLVQTEATNCTSSMLNF
Pa_AtMYB3-like2    NGYGCSNRS-----SDEAFKLCNAKETTNSSKSWCDLLGNDTEGRISNSSMSLALERS--
Pa_AtMYB3-like5    AIKLCNIKE--NTNSIPHDDSEHIVFDMTDVNLL------------ETEAGNCTSAIWSL
```

EAR
suppression domain

```
CfMYB5              -----VSSSASVDPPT---------SLCLSLPGLEPPTTPSSKPVDAQTRKDSPSPSPTP
GbMYBR1             MIESNIDRKLFSLVDD---------YLSVSTELSLGFSGMNCS-----------------
Pa_AtMYB3-like1     I----CDSRERDNGGPVQKHDQFPEYDVFSFFDVRNAENEICC-----------------
Pt_AtMYB3-like2     I----CDSRERDNDSHVQKHDQFPEYDVFSFFDVRNAENEICC-------SADQWVHEQE
Pa_AtMYB4-like3     IKEEDCNSDDPVNSSK---------LWEESFNSINILNSDMENMMFGYLETEVELFGCLA
Pt_AtMYB4-like1     -----KQNVVNSEQGE---------NDLGEGMGYLAYHSGECA-----------------
Pt_AtMYB4-like3     I----LKSKPGEEEGA---------GGRFDEKGYL-FRYEECP-----------------
Pa_AtMYB4-like1     -----EQGESNLSEGK---------GYLCRHGECPPPQSPTDG-----------------
AtMYB3              RRWGSELFGAHESDAV---------CLCCRIGLFRNESCRNCR-----------------
Pt_AtMYB4-like6     ------------------------------------------------------------
AtMYB32             -----RNLRFGRVKYR---------CSACR-FGFGNGKECSCN-------------NVKC
AtMYB7              -----QNQRE---------------ISTCTASRFYMENDMECS----------SETVKCQ
AtMYB4              -----LQGHGKSTTPR---------CFKCS-LGMINGMECRCG-----------------
Pt_AtMYB4-like5     R----LSNDCTQQDHK---------EKICCHIGLRTVGESCCS-----------------
EgMYB1              -----LLRFTGRKSDL---------CXECN-LGLKNSQNCRCS-----------------
Potri_MYB221        -----QAFKTGGSTSL---------CFACS-LGLQNSKDCSCS-----------------
Potri_MYB156        -----QPFKTGGSRSL---------CFACS-LGLQNSKDCSCN-----------------
Pt_AtMYB4-like4     -----RAMDANSNSGL---------GVWCH-TGPQINSDPRCE-----------------
Pa_AtMYB4-like2     ------------------------------------------------------------
Pt_AtMYB4-like2     R----KTVDSNLNSGS---------SEVCYPVGLQINYGKYCD-----------------
ZmMYB31             -----VRPAVKREAGL---------CFGCS-LGLPRTADCKCS-----------------
PvMYB4              AVKREMLQAGHGTLGL---------CFGCSLGLQKGAAGCTCS-----------------
ZmMYB42             VKREALQAGHGHGHGL---------CLGCGLGGQKGAAGCSCS-----------------
Pt_AtMYB3-like6     -----QTDRINLLPPS---------VSPSGSEILQEKQSSSCN-----------------
Pt_AtMYB3-like4     SMTNSSNVTDDQHQHQPQS------PFVSQSIILQENHSSSCD-----------------
Pa_AtMYB3-like4     -----SAVQLPPQLSV---------NLSCPRFIFDLPQRSHTD--SKKKQITMDGAFEIE
Pt_AtMYB3-like1     -----CGTDYDNQFPS---------PTLGRDILLQENGASSCN-----------------
Pt_AtMYB3-like3     IQDFSLESLLLPSVYN---------FDEICPVQSQLNSSDHVSCFAVAAEQAMTDIYNNT
Pa_AtMYB3-like3     -----IHFDY---------------KFSCQNSADDLFQAGNCS-----------------
Pt_AtMYB3-like5     GDQQSPYSDYFEGDAA---------NLAESLLFLTDLSSPDCN-------LLASPSDCST
Pa_AtMYB3-like2     -----PNSYYFGTDAA---------TLADSLLDLNELSSPDCS-------LLSPPSNCSA
Pa_AtMYB3-like5     EEERSPHSYYFAVDTA---------TLDESLSELNVLSSPDCN-----------------


CfMYB5              VPTVPVPVPVQVQIPVPASAFGYVRA--------DEAMSWMSAVVRATVAHTLAPILNSP
GbMYBR1             -----VSKFSTNSHNLYLMGSSLSNT--------DHHMPCGDQSVCRDQYRWMNT-----
Pa_AtMYB3-like1     NDDQWVHEQEMPQLHSWDNQIDDQGK--------EHFGSHVNNDVTAMSWEASFWF----
Pt_AtMYB3-like2     AEFMQQKDQEMAQLGSWEKQIDDQEK--------ENFESHVNNDVTAMSWEASFWF----
Pa_AtMYB4-like3     DPPMPAPNADEGNMETLSSDADYNTE--------DELWSTFLSNSEKTTLPSPSSLSA--
Pt_AtMYB4-like1     --------------------PPMLLL--------R-------------------------
Pt_AtMYB4-like3     -----------------------VGK--------SLLR----------------------
Pa_AtMYB4-like1     ------------------------------------------------------------
AtMYB3              -------------------VSDVRTH----------------------------------
Pt_AtMYB4-like6     ------------------------------------------------------------
AtMYB32             QTEDSSSSSYSSTDISSSIGYDFLGL--------NNTRV--------LDFSTLEMK----
AtMYB7              TENSSSISYSSIDISSSNVGYDFLGL--------KTRI---------LDFRSLEMK----
AtMYB4              -RMRCDVVGGSSKGSDMSNGFDFLGL--------AKKETTSL-----LGFRSLEMK----
Pt_AtMYB4-like5     --------------GCWD------------------------------------------
EgMYB1              ---------VGVIESETSVGYDFLGL--------KASV---------LDYRS--------
Potri_MYB221        ---VIVGTIGSSSSAGSKTGYDFLGM--------KSGV---------LDYRGLEMK----
Potri_MYB156        ---VIVSTVGSSGSTSTKTGYDFLGM--------KSGV---------LDYRSLEMK----
Pt_AtMYB4-like4     ------------NRYCERNSFTELDS--------SSGSIHGILD---LQLSALGVQ----
Pa_AtMYB4-like2     --------------------ESFQRL----------------------------------
Pt_AtMYB4-like2     ------------SGYCEQNASCFSQF--------RLVL----------------------
ZmMYB31             -------------------SSSFLGL--------RTAM---------LDFRSLEMK----
PvMYB4              ------------------SNSHFLGL--------RVGML--------LDFRGLEMK----
ZmMYB42             ------------------NGHHFLGL--------RTSV---------LDFRGLEMK----
Pt_AtMYB3-like6     -----------------LLEEEFLQT--------ENFNSTYF------------------
Pt_AtMYB3-like4     -LIQQESPHSKDFISSYFSSFLDLGE--------QSILLP--------------------
Pa_AtMYB3-like4     NLELLDQEKGRNQDWVCQGGESHDGT--------TSQEQRGMPRDLGRAESRSQLQDHGS
Pt_AtMYB3-like1     -FFDQEFPHSKEFISPYSDNCLDLGE--------PSFVIP--------------------
Pt_AtMYB3-like3     LEPDWLTPVEYEKQSTQSDQMNLLGNFFLSEENWEEEVPTSLSKIQEEEYRAILVDISNK
Pa_AtMYB3-like3     ------ASDCSETEILQQLLHPCSDM--------EEYWREVHGDAQLFAFDSVWNMV---
Pt_AtMYB3-like5     DFASEDLYRGPAALPEETNGFEDMCS--------DQNNMQRVNSS--SMDQELEGFYNNA
Pa_AtMYB3-like2     DFGLQDFCGEPVTLTAEANEFEVVSS--------DQSIMQRDKGFTVLDDQGIEEFYDHT
Pa_AtMYB3-like5     ------LLYSASGSSTDFELEEFYRE--------AATLATGINDSVFT------------
```

```
CfMYB5              PPRGSASDHG-------------------------------------
GbMYBR1             -----------------------------------------------
Pa_AtMYB3-like1     -----------------------------------------------
Pt_AtMYB3-like2     -----------------------------------------------
Pa_AtMYB4-like3     -----------------------------------------------
Pt_AtMYB4-like1     -----------------------------------------------
Pt_AtMYB4-like3     -----------------------------------------------
Pa_AtMYB4-like1     -----------------------------------------------
AtMYB3              -----------------------------------------------
Pt_AtMYB4-like6     -----------------------------------------------
AtMYB32             -----------------------------------------------
AtMYB7              -----------------------------------------------
AtMYB4              -----------------------------------------------
Pt_AtMYB4-like5     -----------------------------------------------
EgMYB1              -----------------------------------------------
Potri_MYB221        -----------------------------------------------
Potri_MYB156        -----------------------------------------------
Pt_AtMYB4-like4     -----------------------------------------------
Pa_AtMYB4-like2     -----------------------------------------------
Pt_AtMYB4-like2     -----------------------------------------------
ZmMYB31             -----------------------------------------------
PvMYB4              -----------------------------------------------
ZmMYB42             -----------------------------------------------
Pt_AtMYB3-like6     -----------------------------------------------
Pt_AtMYB3-like4     -----------------------------------------------
Pa_AtMYB3-like4     RSSPS------------------------------------------
Pt_AtMYB3-like1     -----------------------------------------------
Pt_AtMYB3-like3     -----------------------------------------------
Pa_AtMYB3-like3     -----------------------------------------------
Pt_AtMYB3-like5     QDEDWIHELDCLENSGAQPLSSLLLESEDEWEERSIGKLALEDHVQI
Pa_AtMYB3-like2     QPLDWIHELDYIEKSSPRALSFLLLESEDEREEKLGDHTQIPDVV--
Pa_AtMYB3-like5     -----------------------------------------------
```
