# Supplementary material for: Lignin biosynthesis pathway repressors in gymnosperms: differential repressor domains as compared to angiosperms
Source: For Res (Fayettev). 2024 Sep 19;4:e031. doi: 10.48130/forres-0024-0029 (PMC11524278; doi:10.48130/forres-0024-0029)
Supplement: Supplementary file 1 — Supplementary data to this article can be found online. [file forres-0024-0029-S1.zip › 10.48130_forres-0024-0029-Suppl-TableS1.pdf]

Table S1 List of sequences included in the alignments and phylogenetic tree

Species	TAIR/GenBank/ PopGenIE/ConGenIE/ Gymno Plaza ID	No. of amino acids	Abbreviation	Function	Domain information
Arabidopsis thaliana	AT1G22640	257	AtMYB3	Overexpression downregulates lignin pathway	Contains R2 domain, R3 domain, bHLH interacting domain. Contains provisional hypothetical protein (PLN03091) with interval of 1-131aa. **E-value**: 3.14533e-71. **Contains** EAR domain.
	AT4G38620	282	AtMYB4	Overexpression downregulates lignin pathway	Contains R2 domain, R3 domain, bHLH interacting domain. Contains provisional hypothetical protein (PLN03091) with interval of 1-140 aa. **E-value**: 3.94647e-72. **Contains** EAR domain.
	AT3G13540	249	AtMYB5	Acts as a negative regulator of trichome branching	Contains R2 domain, R3 domain, bHLH interacting domain. Contains MYB5 transcription repressor domain (PLN03212) with interval of 1-249 aa. **E-value**: 0. **Lacks** EAR domain.
	AT2G16720	269	AtMYB7	Suppressor of lignin pathway	Contains R2 domain, R3 domain, bHLH interacting domain. Contains provisional hypothetical protein (PLN03091) with interval of 1-133 aa. **E-value**: 1.03262e-73. **Contains** EAR domain.
	AT4G34990	274	AtMYB32	Suppressor of lignin pathway	Contains R2 domain, R3 domain, bHLH interacting domain. Contains provisional hypothetical protein (PLN03091) with interval of 1-161 aa. **E-value**: 1.3486e-75. **Contains** EAR domain.
	AT1G16490	274	AtMYB58	Positively regulates lignin synthesis	Contains R2 domain, R3 domain, bHLH interacting domain. Contains provisional hypothetical protein (PLN03091) with interval of 4-149 aa. **E-value**: 1.00702e-56. **Lacks** EAR domain.
	AT1G79180	327	AtMYB63	Positively regulates lignin synthesis	Contains R2 domain, R3 domain, bHLH interacting domain. Contains provisional hypothetical protein (PLN03091) with interval of 1-440 aa. **E-value**: 9.98502e-52. **Lacks** EAR domain.
Populus tomentosa (Populus)	KY114929	441	Pto_MYB170	Positively regulates lignin synthesis	Contains R2 domain, R3 domain, bHLH interacting domain. Contains provisional hypothetical protein (PLN03091) with interval of 4-272 aa. **E-value**: 0. **Lacks** EAR domain.
	JQ801749	428	Pto_MYB216	Positively regulates lignin synthesis	Contains R2 domain, R3 domain, bHLH interacting domain. Contains provisional hypothetical protein (PLN03091) with interval of 1-427 aa. **E-value**: 0. **Lacks** EAR domain.
Populus trichocarpa (Populus)	XP_002312966.1	271	Potri_MYB156	Represses the lignin biosynthesis	Contains R2 domain, R3 domain, bHLH interacting domain. Contains provisional hypothetical protein (PLN03091) with interval of 1-131aa. **E-value**: 1.23545e-71. **Contains** EAR domain.
	XP_002306180.1	268	Potri_MYB221	Represses the lignin biosynthesis	Contains R2 domain, R3 domain, bHLH interacting domain Contains provisional hypothetical protein (PLN03091) with interval of 1-131aa. **E-value**: 2.11766e-71. **Contains** EAR domain.
Eucalyptus gunnii (Eucalyptus)	CAE09058.1	255	EgMYB1	Represses the lignin biosynthesis	Contains R2 domain, R3 domain, bHLH interacting domain. Contains provisional hypothetical protein (PLN03091) with interval of 1-163 aa. **E-value**: 2.48018e-70. **Contains** EAR domain.
Panicum virgatum (Switchgrass)	AEM17348.1	258	PvMYB4	Represses the lignin biosynthesis	Contains R2 domain, R3 domain, bHLH interacting domain. Contains provisional hypothetical protein (PLN03091) with interval of 1-151 aa. **E-value**: 7.71002e-75. **Contains** EAR domain.
Zea mays (Maize)	NP_001105949.2	273	ZmMYB31	Represses the lignin biosynthesis	Contains R2 domain, R3 domain, bHLH interacting domain. Contains provisional hypothetical protein (PLN03091) with interval of 1-131 aa. **E-value**: 2.085e-74. **Contains** EAR domain.
	NP_001106009.2	260	ZmMYB42	Represses the lignin biosynthesis	Contains R2 domain, R3 domain, bHLH interacting domain. Contains provisional hypothetical protein (PLN03091) with interval of 1-127 aa. **E-value**: 3.13331e-69. **Contains** EAR domain.
Ginkgo biloba	AWI63367.1	306	GbMYBR1	Overexpression downregulates lignin pathway	Contains R2 domain, R3 domain, bHLH interacting domain. and MYB5 transcription repressor domain (PLN03212) with interval of 4-130 aa. **E-value**: 4.05741e-55. **Lacks** EAR domain.

Species	TAIR/GenBank/ PopGenIE/ConGenIE/ Gymno Plaza ID	No. of amino acids	Abbreviation	Function	Domain information
Picea abies (Norway spruce)	MA_130918p0010	382	Pa_AtMYB3-like1	Shade<Sun at latitude North	Contains R2 domain, R3 domain, bHLH interacting domain. Contains provisional MYB5 transcription repressor domain (PLN03212) with interval of 5-150 aa. **E-value**: 6.07934e-67. **Lacks** EAR domain.
	MA_323706p0010	357	Pa_AtMYB3-like2	Shade<Sun at latitude North	Contains R2 domain, R3 domain, bHLH interacting domain. Contains provisional MYB5 transcription repressor domain (PLN03212) with interval of 4-117 aa. **E-value**: 3.73202e-60. **Lacks** EAR domain.
	MA_10433651p0010	263	Pa_AtMYB3-like3	Shade>Sun at latitude North	Contains R2 domain, R3 domain, bHLH interacting domain. Contains provisional MYB5 transcription repressor domain (PLN03212) with interval of 7-153 aa. **E-value**: 4.12348e-51. **Lacks** EAR domain.
	MA_173020p0010	334	Pa_AtMYB3-like4	Shade North<Shade South	Contains R2 domain, R3 domain, bHLH interacting domain. Contains provisional MYB5 transcription repressor domain (PLN03212) with interval of 4-158 aa. **E-value**: 5.17563e-55. **Lacks** EAR domain.
	MA_7115p0010	293	Pa_AtMYB3-like5	Shade North<Shade South	Contains R2 domain, R3 domain, bHLH interacting domain. Contains provisional MYB5 transcription repressor domain (PLN03212) with interval of 5-116 aa. **E-value**: 6.26073e-56. **Lacks** EAR domain.
	MA_223201p0010	242	Pa_AtMYB4-like1	Shade<Sun at latitude North	Contains R2 domain, R3 domain, bHLH interacting domain. Contains provisional hypothetical protein (PLN03091) with interval of 1-190 aa. **E-value**: 6.84075e-65. **Contains** EAR domain.
	MA_199974p0010	210	Pa_AtMYB4-like2	Shade>Sun at latitude North	Contains R2 domain, R3 domain, bHLH interacting domain . Contains provisional hypothetical protein (PLN03091) with interval of 1-129 aa. **E-value**: 1.36395e-69. **Contains** EAR domain.
	MA_93127p0010	358	Pa_AtMYB4-like3	Shade>Sun at latitude North	Contains R2 domain, R3 domain, bHLH interacting domain. Contains provisional MYB5 transcription repressor domain (PLN03212) with interval of 5-129 aa. **E-value**: 1.10792e-67. **Lacks** EAR domain
Pinus taeda (Loblolly pine)	PITA_000010254	304	Pt_AtMYB3-like1	Shade<Sun at latitude South	Contains R2 domain, R3 domain, bHLH interacting domain. Contains provisional MYB5 transcription repressor domain (PLN03212) with interval of 10-144 aa. **E-value**: 1.62189e-53. C**ontains** EAR domain.
	PITA_000052296	417	Pt_AtMYB3-like2	Shade<Sun at latitude South and North	Contains R2 domain, R3 domain, bHLH interacting domain. Contains provisional MYB5 transcription repressor domain (PLN03212) with interval of 5-136 aa. **E-value**: 1.90929e-66. **Lacks** EAR domain.
	PITA_000074422	531	Pt_AtMYB3-like3	Shade<Sun at latitude South	Contains R2 domain, R3 domain, bHLH interacting domain. Contains provisional MYB5 transcription repressor domain (PLN03212) with interval of 3-118 aa and 184-270 aa. **E-value**: 1.7664e-46 . **Lacks** EAR domain.
	PITA_000071108	312	Pt_AtMYB3-like4	Shade<Sun at latitude South<br>Shade North>Shade South	Contains R2 domain, R3 domain, bHLH interacting domain. Contains provisional MYB5 transcription repressor domain (PLN03212) with interval of 18-183 aa. **E-value**: 3.31219e-57. **Lacks** EAR domain.
	PITA_000078623	400	Pt_AtMYB3-like5	Shade<Sun at latitude South and North<br>Shade North>Shade South	Contains R2 domain, R3 domain, bHLH interacting domain. and contains provisional MYB5 transcription repressor domain (PLN03212) with interval of 30-145 aa. **E-value**: 3.62447e-58. **Lacks** EAR domain.
	PITA_000054044	331	Pt_AtMYB3-like6	Shade>Sun at latitude South<br>Shade North>Shade South	Contains R2 domain, R3 domain, bHLH interacting domain. Contains provisional MYB5 transcription repressor domain (PLN03212) with interval of 72-192 aa. **E-value**: 1.09502e-51. **Lacks** EAR domain.
	PITA_000032473	386	Pt_AtMYB3-like7	Shade>Sun at latitude North	Contains R2 domain, R3 domain, bHLH interacting domain. Contains provisional hypothetical protein (PLN03091) with interval of 1-237 aa. **E-value**: 2.27093e-65. **Lacks** EAR domain.

Species	TAIR/GenBank/ PopGenIE/ConGenIE/ Gymno Plaza ID	No. of amino acids	Abbreviation	Function	Domain information
Pinus taeda (Loblolly pine)	PITA_000047748	243	Pt_AtMYB4-like1	Shade<Sun at latitude South Shade North>Shade South	Contains R2 domain, R3 domain and bHLH interacting domain. Contains provisional MYB5 transcription repressor domain (PLN03212) with interval of 5-152 aa. **E-value**: 1.06152e-64. **Contains** EAR domain.
	PITA_000002313	255	Pt_AtMYB4-like2	Shade>Sun at latitude South and North	Contains R2 domain, R3 domain and bHLH interacting domain. Contains provisional hypothetical protein (PLN03091) with interval of 1-129 aa. **E-value**: 7.13904e-67. **Contains** EAR domain.
	PITA_000008377	245	Pt_AtMYB4-like3	Shade>Sun at latitude South and North	Contains R2 domain, R3 domain and bHLH interacting domain. Contains provisional hypothetical protein (PLN03091) with interval of 1-128 aa. **E-value**: 3.75629e-67. **Contains** EAR domain.
	PITA_000038178	287	Pt_AtMYB4-like4	Shade>Sun at latitude South and North	Contains R2 domain, R3 domain, bHLH interacting domain. Contains provisional hypothetical protein (PLN03091) with interval of 23-203 aa. **E-value**: 2.74522e-62. **Contains** EAR domain.
	PITA_000019252	250	Pt_AtMYB4-like5	Shade>Sun at latitude North	Contains R2 domain, R3 domain, bHLH interacting domain. Contains provisional hypothetical protein (PLN03091) with interval of 1-129 aa. **E-value**: 6.33336e-72. **Lacks** EAR domain.
	PITA_000068957	153	Pt_AtMYB4-like6	Shade>Sun at latitude North	Contains R3 domain, bHLH interacting domain. Contains provisional MYB5 transcription repressor domain (PLN03212) with interval of 14-78 aa. **E-value**: 7.05949e-25. **Lacks** R2 domain and EAR domain.
Cryptomeria fortune (Chinese cedar)	DOI: 10.1016/j.plaphy.2023.107879	313	CfMYB5	Represses the lignin biosynthesis	Contains R2 domain, R3 domain, bHLH interacting domain. Contains provisional hypothetical protein (PLN03091) with interval of 1-164 aa. **E-value**: 1.53e-44. **Contains** EAR domain.
