# Supplementary material for: Lignin biosynthesis pathway repressors in gymnosperms: differential repressor domains as compared to angiosperms
Source: For Res (Fayettev). 2024 Sep 19;4:e031. doi: 10.48130/forres-0024-0029 (PMC11524278; doi:10.48130/forres-0024-0029)
Supplement: Supplementary file 1 — Supplementary data to this article can be found online. [file forres-0024-0029-S1.zip › 10.48130_forres-0024-0029-Suppl-FigureS1.pdf]

**R2 – DNA binding domain is highlighted in yellow.**
**R3 – DNA binding domain is highlighted in green.**
**bHLH binding motif – Indicated by red box.**
**C1 motif – Highlighted in pink.**
**EAR – Suppression domain is highlighted in light blue (which comprises part of the C2 motif).**
**Alternative EAR domain in Pt_AtMYB4-like1, Pt_AtMYB4-like3, Pt_AtMYB4-like4 and Pa_AtMYB4-like1 are marked with box and, bold and underlined.**
**Zinc-finger motif – Highlighted in dark blue (C3 motif).**
**C4 motif – Highlighted in grey. The GY/FDFLGL motif is indicated by black box.**

**Figure S1 Alignment of *Arabidopsis thaliana* MYB3 (AtMYB3), *Gingko biloba* MYB (GbMYBR1) and all MYB3-like copies from *Picea abies* (Pa)**

```
Pa_AtMYB3-like3      MGRPQSCS-----GLKLRKGAWTSEEDLLLKKCIEVHGE-GSWPLLPQKTGLQRSGKSCR
AtMYB3               MGRSPCCE-----KAHMNKGAWTKEEDQLLVDYIRKHGE-GCWRSLPRAAGLQRCGKSCR
Pa_AtMYB3-like4      MGRSPSCSSYSKHEDGLNRGAWTAGEDKILSEYVKTHGV-GRWRSLPKKAGLRRCGKSCR
Pa_AtMYB3-like2      MGRSPCCS-----KEGLNRGAWTKTEDIILCEYIRIHGD-GGWRTLPKKAGLKRCGKSCR
Pa_AtMYB3-like5      MGRS-CCS-----KEGLNRGAWSRKEDMILSEYIRIHGD-GGWTNLPQRAGLKRCGKSCR
GbMYBR1              MGRSPMCS---KVGLGLNKGAWTVEEDNLLIKYFQTHDEGGWKSVPKKAGLKRCGKSCR
Pa_AtMYB3-like1      MGRAPCCA-----KVGLNKGAWSAEEDSLLGKYIQTHGE-GNWRSLPKKAGLRRCGKSCR
                     ***.  *         :..***:  ** :* . .  *.  * *  :*. :**.*.*****

Pa_AtMYB3-like3      LRWLNYLSPNVKRGNISEDEELLIIRMHRLLGNRWALIAGRIPGRSDNEIKNYWNTHLSK
AtMYB3               LRWMNYLRPDLKRGNFTEEEDELIIKLHSLLGNKWSLIAGRLPGRTDNEIKNYWNTHIKR
Pa_AtMYB3-like4      LRWLNYLRPDIKRGNISPEEEELIIRLYRLLGNRWALISGRLPGRTDNEIKNYWNTHLSK
Pa_AtMYB3-like2      LRWLNYLRPDIKRGGISPDEEELIIRLHRLLGNRWSLIAGRLPGRTDNEIKNYWNTCLSK
Pa_AtMYB3-like5      LRWTNYLRPDIKLGNISPDEDELIIRMHRLLGNRWSLIAGRLPGRTDNEIKNYWNTRLSK
GbMYBR1              LRWMNYLRPNIKRGNISPDEEDLIIRLHGLLGNRWALIAGRIPGRTDNEIKNYWYTTLSK
Pa_AtMYB3-like1      LRWLNYLRPCIKRGNITADEEELIIRMHALLGNRWSIIAGRVPGRTDNEIKNYWNTNLSK
                     *** *** * :* *.:: :*: ***.:: ****.*::*:**:***:******** * :..

Pa_AtMYB3-like3      KVNRSRSDD--------------------------FNCNKENVVQTLTSEEGKGGQEIG-
AtMYB3               KLLSRGIDPNSHRLINESVVSPSSLQND--------VVETIHLDFSGPVKPEPVREEIG-
Pa_AtMYB3-like4      KVRMGESDPRFQNLCK-----RRDVPPYPNYDEDIYASQNAPVKTTGV----RYGRAVA-
Pa_AtMYB3-like2      KLLLSMNKSQSKVT-KNVKSTSKSSPVQNR------VFKTTPVKVTTAV---RLSETIR-
Pa_AtMYB3-like5      KL-LSIDDSQSKSTARNLETSSKSPPPPPN-----HVFKTTPIKITTAV---KFSETVG-
GbMYBR1              RVALKGNEAKEHKTYPMKRSRGHSACKQ-------LIMPDSNTKMQDLLSASPTRKELE-
Pa_AtMYB3-like1      KLAVRGIDPKTHKKVTTDSINRASDRFNQRKGGKLYDCSQRSQRLERNVV--RAGQSTGL
                     .:     .

Pa_AtMYB3-like3      A----ENYSSH----SQPKSR-------ISINTDHNQHADM-----------LEEPSTG-
AtMYB3               MVNNCESSGTT-------------------------------------------SEKDYG-
Pa_AtMYB3-like4      ELENFDSYNPD----HVPDTHDGSTQEGLKAADDHVVEIDT---SKSWSQL-LLEHCMGD
Pa_AtMYB3-like2      PKGSMNGYGCS----NRSSDEAFKLCN-AKETTNS---------SKSWCDL-LGNDTEG-
Pa_AtMYB3-like5      PK-RCNGYGRS----NCSSAEAIKLCN-IKENTNSIPHDDSEHIVFDMTDVNLLETEAG-
GbMYBR1              VSTN-QSLSES----VVSNTDDVRTDSNVQSGSPGLQEIRASRI--------FLPSFRGN
Pa_AtMYB3-like1      VIPNVHNLKADLKGQYIAGPREIQSSNSIRSSSPINTPIQPKSNDFMQPQP-VVSSDAGK
                           .                                                   *

Pa_AtMYB3-like3      ---------LTKCQSYRNTPTEQTDLIHFDYKFSCQNSA--DDLFQAGNCSAS----D---
AtMYB3               -----------NEEDWVLNLELSVG---PSYRY--------ESTRKVSVVDSA--------
Pa_AtMYB3-like4      YQYDRLQPNASSSAVQLPPQLSVNLSCPRFIFDLPQRSHTDSKKKQITMDGAFEIEN---
Pa_AtMYB3-like2      ---------RISNSSMSLALERSPN----SYYFGTDAATLADSLLDLNELSSP----D---
Pa_AtMYB3-like5      ---------NCTSAIWSLEEERSPH----SYYFAVDTATLDESLSELNVLSSP----D---
GbMYBR1              Q-------VSSHSELMAPANPMIESNIDRKLFSLVD----DYLSVSTELSLGFSGMN---
Pa_AtMYB3-like1      QTDDSTVYCSSDSAASCALIDHLSSEDDDQYLSLEGNSNECYSHTVAEESGTLKSSNPQT
                               .   EAR – suppression domain           .

Pa_AtMYB3-like3      -----CSETEILQQLLHPCSDMEEYWR-----EVHGDAQLFAFDSV-------------W
AtMYB3               ---------------------ESTRRWGSELFGAHESDAVCLCCRIG-----------LFR
Pa_AtMYB3-like4      -----LE--------LLDQEKGRNQDWVCQG--GESHDGTTSQEQRG-----------MPR
Pa_AtMYB3-like2      -----CS--------LLSPPSNCSADFGLQ---DFCGEPVTLTAEANEFEVVSSDQSIMQR
Pa_AtMYB3-like5      -----CN--------LLYSASGSSTDFELE---EFYREAATLATGIN--------------
GbMYBR1              -----CS--------VSKFSTNSHNLYLMGS--SLSNTDHHMPCGDQ-----SVCRDQYRW
Pa_AtMYB3-like1      HSEAICDSRERDNGGPVQKHDQFPEYDVFSFFDVRNAENEICCNDDQWVHEQEMPQLHSW
```

```
                                                      :

Pa_AtMYB3-like3      NMV---------------------------------------------------------
AtMYB3               NESCRNCRVSDVRTH---------------------------------------------
Pa_AtMYB3-like4      DLGRA-ESRSQLQDHGSRSSPS--------------------------------------
Pa_AtMYB3-like2      DKGFTVLDDQGIEEFYDHTQPLDWIHELDYIEKSSPRALSFLLLESEDEREEKLGDHTQI
Pa_AtMYB3-like5      DSVFT-------------------------------------------------------
GbMYBR1              MNT---------------------------------------------------------
Pa_AtMYB3-like1      DNQIDDQGKEHFGSHVNNDVTAMSWEASFWF-----------------------------


Pa_AtMYB3-like3      ----
AtMYB3               ----
Pa_AtMYB3-like4      ----
Pa_AtMYB3-like2      PDVV
Pa_AtMYB3-like5      ----
GbMYBR1              ----
Pa_AtMYB3-like1      ----
```
